# Connecting Quorum Sensing, c-di-GMP, Pel Polysaccharide, and Biofilm Formation in *Pseudomonas aeruginosa* through Tyrosine Phosphatase TpbA (PA3885)

**DOI:** 10.1371/journal.ppat.1000483

**Published:** 2009-06-19

**Authors:** Akihiro Ueda, Thomas K. Wood

**Affiliations:** 1 Artie McFerrin Department of Chemical Engineering, Texas A & M University, College Station, Texas, United States of America; 2 Department of Biology, Texas A & M University, College Station, Texas, United States of America; 3 Zachry Department of Civil Engineering, Texas A & M University, College Station, Texas, United States of America; Massachusetts General Hospital, United States of America

## Abstract

With the opportunistic pathogen *Pseudomonas aeruginosa*, quorum sensing based on homoserine lactones was found to influence biofilm formation. Here we discern a mechanism by which quorum sensing controls biofilm formation by screening 5850 transposon mutants of *P. aeruginosa* PA14 for altered biofilm formation. This screen identified the PA3885 mutant, which had 147-fold more biofilm than the wild-type strain. Loss of PA3885 decreased swimming, abolished swarming, and increased attachment, although this did not affect production of rhamnolipids. The PA3885 mutant also had a wrinkly colony phenotype, formed pronounced pellicles, had substantially more aggregation, and had 28-fold more exopolysaccharide production. Expression of PA3885 in *trans* reduced biofilm formation and abolished aggregation. Whole transcriptome analysis showed that loss of PA3885 activated expression of the *pel* locus, an operon that encodes for the synthesis of extracellular matrix polysaccharide. Genetic screening identified that loss of PelABDEG and the PA1120 protein (which contains a GGDEF-motif) suppressed the phenotypes of the PA3885 mutant, suggesting that the function of the PA3885 protein is to regulate 3,5-cyclic diguanylic acid (c-di-GMP) concentrations as a phosphatase since c-di-GMP enhances biofilm formation by activating PelD, and c-di-GMP inhibits swarming. Loss of PA3885 protein increased cellular c-di-GMP concentrations; hence, PA3885 protein is a negative regulator of c-di-GMP production. Purified PA3885 protein has phosphatase activity against phosphotyrosine peptides and is translocated to the periplasm. Las-mediated quorum sensing positively regulates expression of the PA3885 gene. These results show that the PA3885 protein responds to AHL signals and likely dephosphorylates PA1120, which leads to reduced c-di-GMP production. This inhibits matrix exopolysaccharide formation, which leads to reduced biofilm formation; hence, we provide a mechanism for quorum sensing control of biofilm formation through the *pel* locus and suggest PA3885 should be named TpbA for tyrosine phosphatase related to biofilm formation and PA1120 should be TpbB.

## Author Summary

Most bacteria live in biofilms, which are complex communities of microorganisms attached to a surface via polysaccharides; these biofilms are responsible for most human bacterial diseases. The pathogen *Pseudomonas aeruginosa* is best-studied for biofilm formation. Currently, it is recognized that cell communication or quorum sensing is important for biofilm formation, but how these external signals are converted into internal signals to regulate the networks of genes that result in biofilm formation is not well understood. Here, by studying 5850 bacterial strains, each of which lacks a single protein, we identify a new enzyme of *P. aeruginosa*, a tyrosine phosphatase (TpbA), that links extracellular quorum sensing signals to polysaccharide production and biofilm formation. We find that TpbA is subject to control by quorum sensing signals, that it is in the periplasm, and that it controls the level of the intracellular secondary messenger 3,5-cyclic diguanylic acid (c-di-GMP). By controlling c-di-GMP concentrations, TpbA serves to regulate biofilm formation, rapid cell movement on the surface, colony morphology, cell aggregation, and polysaccharide production. The importance of our study is that it shows the secondary messenger c-di-GMP may be regulated by tyrosine phosphorylation; hence, it provides a new target for controlling bacterial social behavior.

## Introduction

*Pseudomonas aeruginosa*, an opportunistic pathogen, is often used to elucidate how biofilms form because persistence of this bacterium is linked to its ability to form biofilms [1]. Biofilms are formed by the attachment of bacteria to submerged surfaces in aquatic environments through their production of microbial products including polysaccharides, proteins, and nucleic acids [1]. In *P. aeruginosa* PA14, the glucose-rich extracellular polysaccharide (EPS) of the biofilm matrix is formed by proteins encoded by the *pel* operon; note the related strain *P. aeruginosa* PAO1 has two EPS production loci, *pel* and *psl* [2],[3]. Mutations in the *pel* locus of *P. aeruginosa* PA14 dramatically decrease biofilm formation as well as pellicle formation; pellicles are formed at the interface between the air and liquid medium [3].

Regulation of Pel polysaccharide involves 3,5-cyclic diguanylic acid (c-di-GMP) which is formed by diguanylate cyclases with GGDEF motifs that synthesize this second messenger; phosphodiesterases with EAL motifs catabolize c-di-GMP. Many proteins with GGDEF motifs enhance biofilm formation [4]; for example, c-di-GMP increases cellulose biosynthesis in *Acetobacter xylinus* [5], and c-di-GMP enhances EPS production by binding the PelD protein that is a c-di-GMP receptor in *P. aeruginosa* PA14 [6]. Thus, biofilm formation is controlled by a signal cascade mediated by a complex of c-di-GMP and PelD in *P. aeruginosa* PA14; however, the upstream portions of this cascade have not been elucidated [7].

Quorum sensing (QS) is bacterial communication using diffusible molecules known as autoinducers to regulate population behavior and is related to both polysaccharide production and biofilm formation. To date, three QS systems have been identified in *P. aeruginosa*. Las-based QS is regulated by *N*-(3-oxododecanoyl)-L-homoserine lactone, produced by LasRI [8], and Rhl-based QS is regulated by *N*-butyryl homoserine lactone, produced by RhlRI [9]. The third QS molecule, 2-heptyl-3-hydroxy-4-quinolone (PQS), was identified as a regulator for both Las- and Rhl-QS [10]. These cell communication signals regulate several phenotypes including virulence and antibiotic resistance [11]. Although the relationship between QS and biofilm formation has not been fully elucidated, some lines of evidence show the importance of QS for biofilm formation. Cells lacking Las-QS in *P. aeruginosa* form flat biofilms, and this structural abnormality makes bacteria in biofilms more sensitive to antibiotic treatment [12]. Biofilm architecture is regulated by rhamnolipids whose synthesis is controlled by Rhl-QS [13]. Thus, *P. aeruginosa* QS seems to participate in the development of biofilm architecture rather than initiation of biofilm formation. In addition, LasR- and RhlR-QS have been shown to influence the *pel* operons indirectly and another transcriptional regulator that controls *pel* has been predicted [7].

Protein phosphorylation and dephosphorylation are well-conserved posttranslational modifications in both prokaryotes and eukaryotes [14]. Protein kinases and phosphatases modulate cellular activity by adding and removing phosphate groups at Ser, Thr, or Tyr residues. Phosphorylation also occurs at His and Asp residues by histidine kinases and response regulators in two-component regulatory systems. Although the discovery of protein phosphorylation was delayed in prokaryotes compared to eukaryotes [14], many genome sequences predict the existence of phosphorylation/dephosphorylation systems in prokaryotes. The *P. aeruginosa* genome encodes an extraordinary number of the genes for two-component regulatory systems [15], and diverse cellular functions are regulated by His-Asp phosphorylation including chemotaxis, iron acquisition, alginate production, and virulence factors [16]. In contrast, phosphorylation at Ser, Thr, and Tyr residues has not been studied well in *P. aeruginosa*; although, Fha1 of the Type VI secretion system is posttranslationally regulated through Thr phosphorylation by the protein kinase PpkA and dephosphorylated by the phosphatase PppA [17]. In *Bacillus subtilis*, mutations in *prkC*, a Ser/Thr kinase, and *prpC*, a phosphatase, decrease sporulation and biofilm formation [18]. Mutations in *stk1*, a Ser/Thr kinase, and *stp1*, a phosphatase of Stk1, decrease virulence in *Streptococcus agalactiae* [19]. These findings show that posttranslational modification via protein phosphorylation at Ser, Thr, and Tyr residues regulates various cellular functions.

In this study, our goal was to explore the complex regulatory cascade that includes detection of QS signals, Pel polysaccharide production, and biofilm formation. By screening 5850 transposon mutants for altered biofilm formation, we identified and characterized a novel protein tyrosine phosphatase, TpbA (tyrosine phosphatase related to biofilm formation), that represses biofilm formation through the *pel* locus. The *tpbA* mutant displays pleiotropic phenotypes such as hyperbiofilm formation, enhanced EPS production, altered colony morphology, increased aggregation, elevated c-di-GMP, and abolished swarming. Loss of an uncharacterized GGDEF protein, PA1120 (TpbB), suppressed these phenotypes, indicating that TpbA controls c-di-GMP production through TpbB. Therefore, the mechanism for QS-control of biofilm formation has been extended to include a novel phosphatase (TpbA), a diguanylate cyclase (TpbB), and c-di-GMP; hence, the predicted additional level of control of the *pel* polysaccharide locus has been identified and involves c-di-GMP as controlled by a tyrosine phosphatase.

## Results

### TpbA negatively regulates biofilm formation and positively regulates swimming and swarming

**Figure 1:**
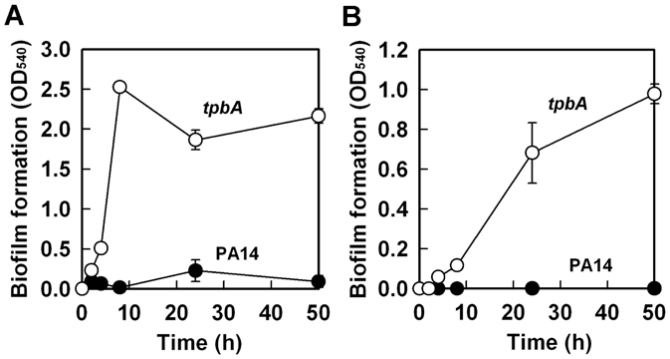
Inactivation of *tpbA* increases biofilm formation. Total biofilm formation (at the liquid/solid and air/liquid interfaces) (A), and biofilm formation on the bottom of polystyrene plates (B) by *P. aeruginosa* PA14 and the *tpbA* mutant at 37°C in LB after 50 h. Six to ten wells were used for each culture. Data show the average of the two independent experiments±s.d.

Previously, by screening 5850 transposon mutants for altered biofilm formation, we identified 137 transposon mutants of *P. aeruginosa* PA14 with over 3-fold enhanced biofilm formation [20]. Among these mutants, the *tpbA* (PA3885) mutant increased biofilm formation by 147-fold after 8 h in LB medium at 37°C (Fig. 1A). This significant increase in biofilm formation upon inactivating *tpbA* is partially due to enhanced attachment to the polystyrene surface because biofilm formation at the bottom of the plates (solid/liquid interface) increased gradually with the *tpbA* mutant while PA14 did not form biofilm on the bottom of the plate (Fig. 1B).

**Figure 2:**
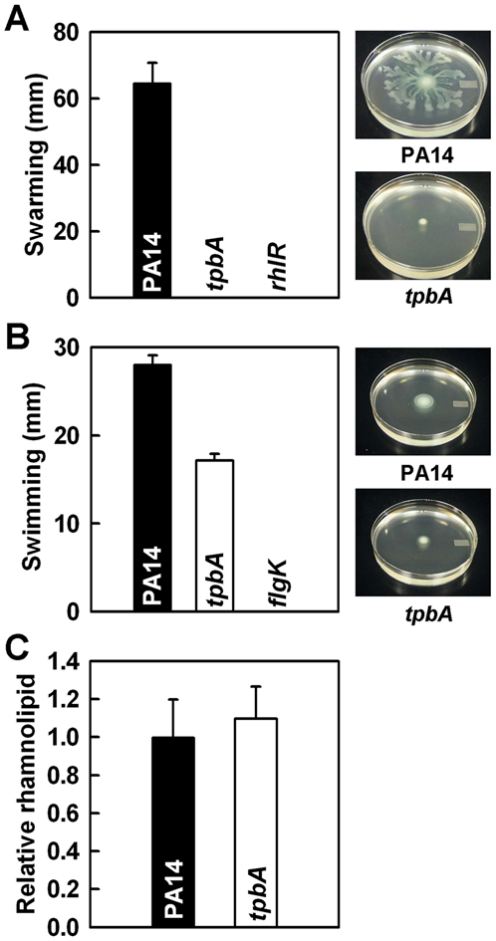
TpbA regulates swarming, swimming motility, and production of rhamnolipids. Swarming motility (A), swimming motility (B), and production of rhamnolipids (C) of *P. aeruginosa* PA14 and the *tpbA* mutant at 37°C after 24 h. Five plates were used for each swarming and swimming culture, and data show the average of two independent experiments. For the production of rhamnolipids, data show the average of the two independent experiments±s.d.

Motility often influences biofilm formation in *P. aeruginosa*; biofilm formation is inversely influenced by swarming motility [21], and swimming motility increases initial attachment to surfaces during biofilm development [22]. To examine the relationship between enhanced biofilm formation and motility in the *tpbA* mutant, we examined swimming and swarming motility for this mutant; *rhlR* [23] and *flgK* [22] mutants were used as negative controls for swarming and swimming motility, respectively. Although PA14 swarmed on the surface of plates at 24 h, the *tpbA* mutations abolished swarming like the *rhlR* mutation (Fig. 2A). The *tpbA* mutation also decreased swimming motility by 40% (Fig. 2B). Swarming is positively influenced by production of the biosurfactant putisolvins in *P. putida* [24] and rhamnolipids in *P. aeruginosa* [23]. However, no significant difference was found in the production of rhamnolipids between PA14 and the *tpbA* mutant (Fig. 2C). This shows the *tpbA* mutation abolishes swarming in a manner distinct from the production of rhamnolipids.

### TpbA affects colony morphology, decreases EPS, and decreases pellicle production

**Figure 3:**
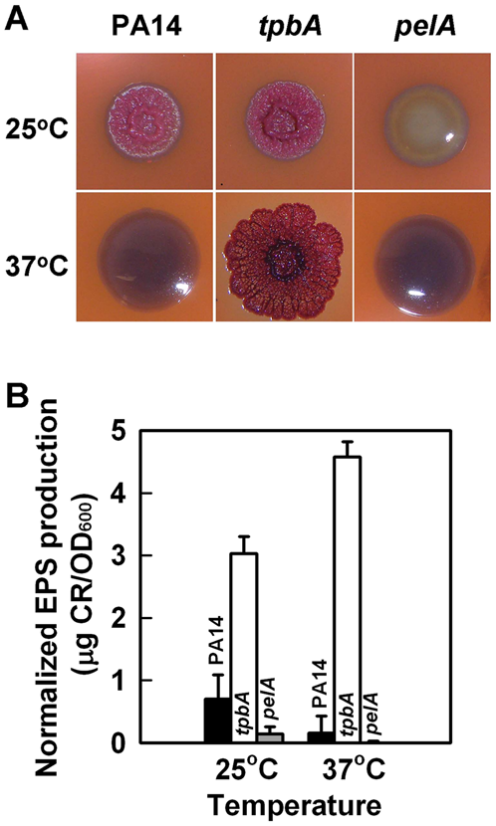
Inactivation of *tpbA* increases colony roughness and enhances EPS production. Colony morphology of *P. aeruginosa* PA14, the *tpbA* mutant, and the *pelA* mutant on Congo-red plates after 6 days at 25°C or 37°C (A). EPS production of each strain after 24 h at 37°C or after 48 h at 25°C (B). Data show the average of the two independent experiments±s.d.

Congo-red is often used to observe colony morphology because it detects EPS production and this impacts biofilm formation; for example, the *wspF* mutant shows wrinkly colony morphology on Congo-red plates and has increased biofilm formation [25], while smooth colonies like the *pelA* mutant [3] form less biofilm. We found that the *tpbA* mutant formed a red, wrinkly colony when it was grown on Congo-red plates at 37°C, although PA14 and the *pelA* mutant formed white smooth colonies (Fig. 3A). When the bacteria were grown at 25°C, both PA14 and the *tpbA* mutant formed red wrinkly colonies, but the *pelA* mutant still formed a white smooth colony (Fig. 3A). These observations with the *pelA* mutant and wild-type PA14 are identical to the previous report that expression of the *pel* genes is induced at room temperature and repressed at 37°C [7]. Therefore, the red wrinkly colony formed by the *tpbA* mutant at 37°C implies increased production of EPS via Pel.

We also quantified the amount of EPS bound to cells of PA14 and the *tpbA* mutant at both 37°C and 25°C. As shown in Fig. 3B, the *tpbA* mutant produced 28-fold more EPS than PA14 at 37°C. The *tpbA* mutant also produced 4.3-fold more EPS than PA14 at room temperature. The *pelA* mutant (negative control) did not form EPS at both temperatures tested. We also found that the *tpbA* mutant formed a pronounced pellicle at 37°C after 1 day, but PA14 and the *pelA* mutant did not form a pellicle (data not shown). At 25°C, both the *tpbA* mutant and PA14 formed pellicles after 5 days. Taken together with the EPS production data, TpbA reduces pellicle formation by decreasing Pel activity.

### Differentially regulated genes in biofilm cells of the *tpbA* mutant

To confirm the impact of the *tpbA* mutation on *pel* expression and to investigate its impact on the whole genome, a whole-transcriptome analysis was performed with biofilm cells of the *tpbA* mutant at 37°C at 7 h; planktonic cells were not assayed since we were primarily interested in how TpbA controls biofilm formation. Inactivation of *tpbA* altered diverse loci including genes related to EPS production (*pelACDF* induced approximately 4-fold), transport (PA2204 repressed approximately 5-fold, PA4142–PA4143 induced approximately 3-fold), type IV pili (PA4302 to PA4306 repressed approximately 4-fold), and a putative adhesin and its regulator (PA4624–PA4625 induced approximately 4-fold) (Tables S1 and S2). Expression of *tpbA* was induced as much as 120-fold in the *tpbA* mutant, suggesting that TpbA negatively regulates its transcription. The whole-transcriptome experiments were performed twice using independent cultures of PA14 and the *tpbA* mutant at 7 h, and most of the differentially regulated genes were consistently altered except *pel* genes which were induced the most in the samples containing an RNase inhibitor. A whole-transcriptome analysis was also conducted using biofilm cells at 4 h since the mode of growth switched from planktonic to biofilm for the *tpbA* mutant at this time (Fig. 1A). Similar to the 7 h results, several loci were induced including *pelAEF* (1.5- to 1.7-fold), *tpbA* (42-fold), PA1168–PA1169 (1.4- to 2.1-fold), PA3886 (3.5-fold), and PA4624–PA4625 (2- to 3.7-fold) (Table S1).

To verify induction of the *pel* locus, expression of *pelA* was determined by quantitative real time-PCR (qRT-PCR). Using two independent RNA samples extracted from biofilm cells at 7 h, *pelA* was induced 112±100-fold in the *tpbA* mutant vs. PA14. These results showed EPS production is induced significantly in the *tpbA* mutant due to overexpression of *pel* genes. qRT-PCR also confirmed induction of PA4625 (7±7-fold) as well as PA4139 (38±30-fold) that encodes a hypothetical protein.

### TpbA represses adhesin expression and reduces aggregation

**Figure 4:**
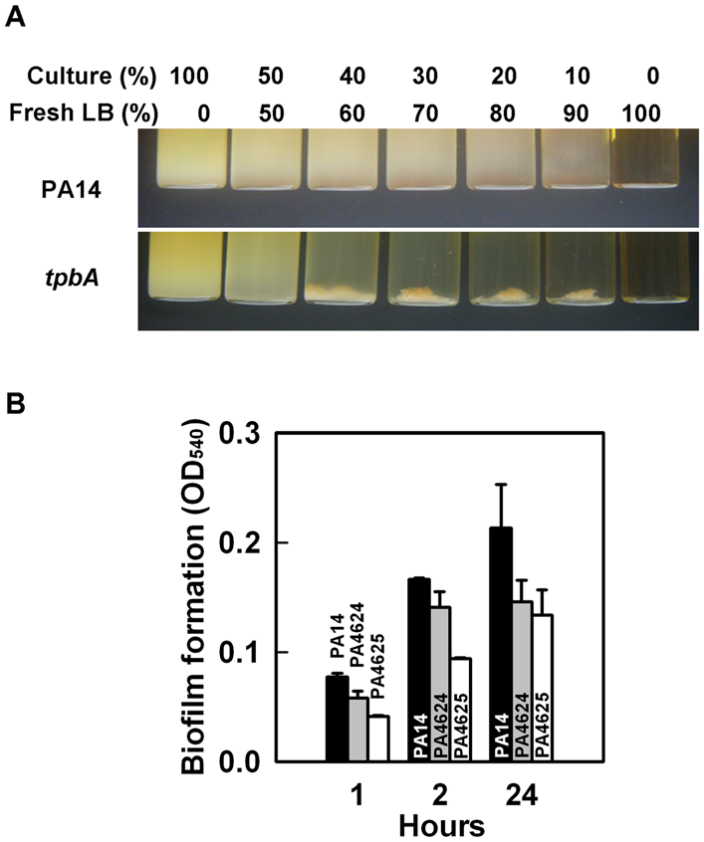
Inactivation of *tpbA* increases cell aggregation. Aggregation of PA14 and the *tpbA* mutant after diluting with fresh LB medium (percentages indicate volume % of the starting overnight culture and fresh medium) (A). Biofilm formation of mutants lacking adhesin (PA4625) and its regulator (PA4624) at 37°C after 1, 2, and 24 h (B). Ten wells were used for each culture. Data show the average of the two independent experiments±s.d.

Cell aggregative behavior is also related to biofilm formation so we investigated the role of TpbA on cell aggregation and found the *tpbA* mutant causes cell aggregation (Fig. 4A). Autoaggregation of the *tpbA* mutant was also observed in 96-well polystyrene plates during biofilm formation (data not shown). Our whole-transcriptome analysis showed that inactivating *tpbA* induced both PA4624 (encodes for a putative hemolysin activator) and PA4625 (encodes for an adhesin/hemagglutinin) by 2.1- to 4.9-fold. In *E. coli*, adhesin regulates cell aggregation as well as attachment [26]. To examine whether PA4624–PA4625 control adhesive activity in *P. aeruginosa*, we investigated biofilm formation with these mutants. Both mutants showed decreased initial biofilm formation; i.e., initial attachment, to polystyrene plates at 1 h and 2 h (Fig. 4B), and final biofilm formation at 24 h was also decreased for both the PA4624 and PA4625 mutants, which suggests that both gene products control attachment to the surface. Therefore, TpbA decreases cell aggregation probably by repressing the PA4624 and PA4625 genes.

### Complementation of the *tpbA* mutation

To verify whether the phenotypes observed in the *tpbA* mutant were caused by loss of function of TpbA, we confirmed transposon insertion in *tpbA* by PCR at residue 25. Furthermore, biofilm formation for both PA14 and the *tpbA* mutant were examined with *tpbA* expressed in *trans* under the control of an arabinose-inducible promoter. *tpbA* expression reduced biofilm formation of the *tpbA* mutant by 33% (Fig. S1A) and abolished biofilm formation on the bottom of the plates (Fig. S1B). Similar results were found upon expressing *tpbA* in wild-type PA14 (OD_540_ value was 0.22±0.02 for PA14/pMQ70 and 0.02±0.01 for PA14/pMQ70-*tpbA*, Fig. S1A). In addition, the aggregative phenotype of the *tpbA* mutant was also complemented by expression of *tpbA* in *trans* (Fig. S1C). Taken together, TpbA functions as a negative regulator of biofilm formation and aggregation in PA14.

### Genetic screening identified Pel and GGDEF-proteins downstream of TpbA

**Figure 5:**
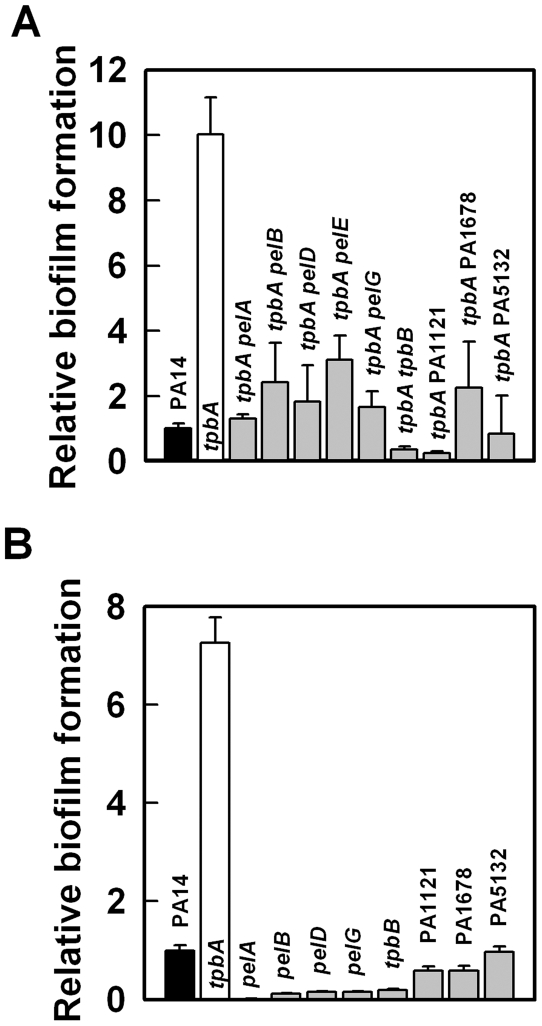
Reduction in biofilm formation by *tpbA* phenotype reversal mutations. Biofilm formation of double mutants (A) and single mutants (B) identified by genetic screening for the *tpbA* mutation at 37°C in LB after 24 h. Six to ten wells were used for each culture. Representative data are shown in (A). Biofilm formation of each mutant was calculated relative to that of PA14 (OD_540_ mutant/OD_540_ wild-type). Data show the average of the two independent experiments±s.d.

To investigate how TpbA regulates biofilm formation, EPS production, wrinkly colony morphology, and cell aggregation, genetic screening was conducted using Tn*5-luxAB* transposon mutagenesis to find suppressive loci for the phenotypes of the *tpbA* mutation. The double mutant library (*tpbA* plus random gene inactivations) was screened first for a reduction in aggregation; this step eliminated most cells with unaltered phenotypes by allowing them to aggregate and precipitate at the bottom of the tube. The cells remaining in the supernatant that failed to aggregate like the *tpbA* mutant were grown on Congo-red plates, incubated at 37°C for 3–4 days, and colonies displaying a white and smooth shape like the wild-type strain were chosen. After that, a third screen was performed by assaying biofilm formation using 96-well polystyrene plates to identify double mutants that had biofilm formation like the wild-type strain. Twenty-six mutants were identified that showed reduced aggregation, a white smooth colony, and reduced biofilm formation like the wild-type strain, and 19 of these mutations were in the *pel* locus (Fig. 5A, Table 1). Four of the other mutants have the Tn*5-luxAB* insertion in the TpbB gene (encodes a GGDEF-motif protein) and in the PA1121 gene (encodes a hypothetical protein). In addition, insertions were found in the PA1678 gene (encodes a putative DNA methylase) and in the promoter of the PA5132 gene (encodes a putative protease) (Table 1). Like the double mutants, all of the single mutants lacking the gene identified by genetic screening were tested for biofilm formation, and all of these mutants formed less biofilm as reported previously (Fig. 5) [3],[4].

**Table 1:** Phenotype reversal loci for the *tpbA* mutation.

Strain	PAO1 ID	PA14 ID	Gene Name	Gene function	Relative biofilm formation	Colony morphology	Aggregation
PA14	-	-	-	-	0.14	white smooth	−
*tpbA*	PA3885	PA14_13660	*tpbA*	Tyrosine phosphatase (this study)	1.00	red wrinkly	+
Mutant 1	PA1120	PA14_49890	*tpbB*	c-di-GMP cyclase, GGDEF motif	0.13	white smooth	−
Mutant 19	PA1120	PA14_49890	*tpbB*	c-di-GMP cyclase, GGDEF motif	0.10	white smooth	−
Mutant 4	PA1121	PA14_49880		Hypothetical protein	0.22	white smooth	−
Mutant 11	PA1121	PA14_49880		Hypothetical protein	0.07	white smooth	−
Mutant 2	PA1678	PA14_42790		Putative DNA methylase	0.30	white smooth	−
Mutant 6	PA1678	PA14_42790		Putative DNA methylase	0.28	white smooth	−
Mutant 5	PA3058	PA14_24560	*pelG*	Predicted membrane protein related to EPS production, PelG	0.24	white smooth	−
Mutant 7	PA3058	PA14_24560	*pelG*	Predicted membrane protein related to EPS production, PelG	0.19	white smooth	−
Mutant 9	PA3058	PA14_24560	*pelG*	Predicted membrane protein related to EPS production, PelG	0.27	white smooth	−
Mutant 12	PA3058	PA14_24560	*pelG*	Predicted membrane protein related to EPS production, PelG	0.25	white smooth	−
Mutant 18	PA3058	PA14_24560	*pelG*	Predicted membrane protein related to EPS production, PelG	0.42	white smooth	−
Mutant 10	PA3060	PA14_24530	*pelE*	Sucrose synthase related to EPS production, PelE	0.41	white smooth	−
Mutant 15	PA3060	PA14_24530	*pelE*	Sucrose synthase related to EPS production, PelE	0.45	white smooth	−
Mutant 3	PA3061	PA14_24510	*pelD*	L-lactate permease related to EPS production, PelD	0.20	white smooth	−
Mutant 16	PA3063	PA14_24490	*pelB*	Conserved hypothetical protein related to EPS production, PelB	0.31	white smooth	−
Mutant 21	PA3063	PA14_24490	*pelB*	Conserved hypothetical protein related to EPS production, PelB	0.21	white smooth	−
Mutant 22	PA3063	PA14_24490	*pelB*	Conserved hypothetical protein related to EPS production, PelB	0.17	white smooth	−
Mutant 23	PA3063	PA14_24490	*pelB*	Conserved hypothetical protein related to EPS production, PelB	0.38	white smooth	−
Mutant 25	PA3063	PA14_24490	*pelB*	Conserved hypothetical protein related to EPS production, PelB	0.28	white smooth	−
Mutant 27	PA3063	PA14_24490	*pelB*	Conserved hypothetical protein related to EPS production, PelB	0.23	white smooth	−
Mutant 8	PA3064	PA14_24480	*pelA*	Oligogalacturonide lyase related to EPS production, PelA	0.18	white smooth	−
Mutant 14	PA3064	PA14_24480	*pelA*	Oligogalacturonide lyase related to EPS production, PelA	0.45	white smooth	−
Mutant 17	PA3064	PA14_24480	*pelA*	Oligogalacturonide lyase related to EPS production, PelA	0.29	white smooth	−
Mutant 20	PA3064	Pro-PA14_24480	*pelA*	Oligogalacturonide lyase related to EPS production, PelA	0.25	white smooth	−
Mutant 24	PA3064	PA14_24480	*pelA*	Oligogalacturonide lyase related to EPS production, PelA	0.24	white smooth	−
Mutant 13	PA5132	Pro-PA14_67780		Putative protease	0.15	white smooth	−

Genetic screening identified additional mutations that mask the phenotypes of the *tpbA* mutant (enhanced biofilm formation, EPS production, wrinkly colony morphology, and aggregation). Relative biofilm formation is shown as a ratio of that of the *tpbA* mutant.

### TpbA negatively controls cellular c-di-GMP concentrations

Results of genetic screening and the whole-transcriptome analysis implied TpbA regulates c-di-GMP concentrations since loss of one of the GGDEF proteins (TpbB) masked the phenotypes of the *tpbA* mutant. *tpbB* encodes a functional GGDEF protein whose activity was confirmed by overexpressing this gene in *P. aeruginosa* [4]. We also confirmed that expression of *tpbB* increases cell aggregation and attachment to tubes so the *tpbB* mutation may be complemented (Fig. S2). In addition, we measured the cellular c-di-GMP concentrations of PA14 and the *tpbA* mutant using high performance liquid chromatography (HPLC) as reported previously [4]. The peaks corresponding to c-di-GMP were observed with the extracts of the *tpbA* mutant, but not with those of PA14, and the peak was confirmed by comparing the spectrum to purified c-di-GMP as well as by spiking the samples with purified c-di-GMP (Fig. S3). We estimated the cellular c-di-GMP concentration was 10±2 pmol/mg cells in the *tpbA* mutant. This is comparable to the c-di-GMP concentration found for a small colony variant that showed aggregation (around 2.0 pmol/mg cells) and a mutant with wrinkly colony morphology [27]. Overexpression of *tpbB* results in c-di-GMP concentrations of 134 pmol/mg cells in PA14 [4]. Therefore, TpbA reduces c-di-GMP concentrations in the cell and probably does so via TpbB.

### TpbA is a tyrosine phosphatase

**Figure 6:**
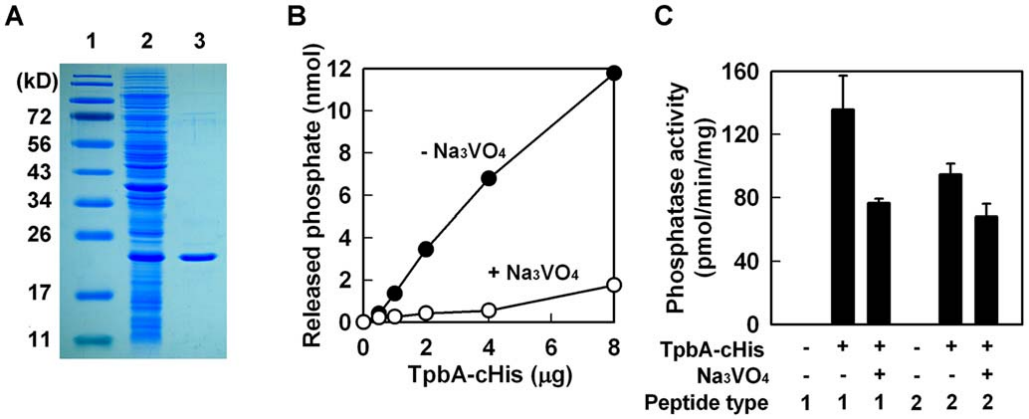
TpbA has phosphatase activity against tyrosine residues. Purification of TpbA-cHis (lane 1: protein marker, lane 2: whole cell lysate from *E. coli* BL21(DE3)/pET28b-13660c after 3 h of IPTG induction, lane 3: purified TpbA-cHis) (A). *p*-Nitrophenyl phosphate phosphatase assay with TpbA-cHis protein (B). Phosphatase reaction was performed at 37°C for 1 h with the indicated amount of protein. Na_3_VO_4_ (10 mM) was used as an inhibitor specific for tyrosine phosphatases. Protein tyrosine phosphatase assay with TpbA-cHis (C). Phosphatase reaction was performed with synthetic phosphotyrosine peptides (type I: END(pY)INASL and type II: DADE(pY)LIPQQG) at 37°C for 3 h. Na_3_VO_4_ (50 mM) was used as an inhibitor.

*tpbA* encodes a 218 aa protein that has the conserved domain for a protein tyrosine phosphatase [28],[29] since it has the C(X)_5_R(S/T) motif beginning at aa 132 ( CKHGNNRT ). To confirm it is a tyrosine phosphatase, we purified TpbA by adding a polyhistidine tag at either the N-terminus (TpbA-nHis) or the C-terminus (TpbA-cHis) (note only the C-terminus fusion protein was active). Expression of recombinant TpbA was confirmed in *E. coli* by clear expression of a band at 24 kD (Fig. 6A). The purified TpbA protein had phosphatase activity with *p*-nitrophenyl phosphate (pNPP) that is often used as a general phosphatase substrate [29] (Fig. 6B). Further proof that TpbA is a tyrosine phosphatase was found using a tyrosine phosphatase specific inhibitor, trisodium orthovanadate [30], that completely inhibited the phosphatase activity of TpbA-cHis (Fig. 6B). The third and fourth lines of evidence that TpbA is a tyrosine phosphatase were found using tyrosine specific substrates; TpbA-cHis dephosphorylated both phosphotyrosine peptides, END(pY)INASL (peptide type I) and DADE(pY)LIPQQG (peptide type II) (Fig. 6C), and this activity was inhibited by trisodium orthovanadate. These results show conclusively that TpbA encodes a tyrosine phosphatase.

### Tyrosine phosphorylation enhances biofilm formation in PA14

To see the effect of tyrosine phosphorylation on biofilm formation, biofilm formation was examined in PA14 with trisodium orthovanadate at 37°C for 4 h which should reduce dephosphorylation by TpbA. Trisodium orthovanadate increased PA14 biofilm formation 3.6-fold (Fig. S4), showing that cellular tyrosine phosphorylation increases biofilm formation.

### TpbA is found in the periplasm

The N-terminal region of TpbA protein has a putative signal peptide, predicted by pSORT [31], that appears necessary for secretion of this protein (28 aa, MHRSPLAWLRLLLAAVLGAFLLGGPLHA). This implied that processing of N-terminal region of TpbA protein may be essential for full phosphatase activity. To prove that TpbA has an active signal sequence, we expressed TpbA in *E. coli* and collected the proteins from cytosolic, periplasmic, and membrane fractions. All fractioned proteins were analyzed by SDS-PAGE, and we found that TpbA exclusively localized in the periplasm (data not shown). Hence, TpbA probably dephosphorylates its substrate in the periplasm which explains why phosphatase activity was seen only with the fusion protein with the His tag at the C-terminus.

### Y48 and Y62 are responsible for TpbB activity

Since TpbA is a tyrosine phosphatase that is found in the periplasm and since TpbB has three likely periplasmic tyrosines (Y48, Y62, and Y95) [32], we mutated the periplasmic tyrosine residues by converting them to phenylalanine and checked for TpbB activity in the *tpbB* mutant. Active TpbB, from overexpression of *tpbB* using the *tpbB* mutant and pMQ70-*tpbB*, always leads to aggregation whereas the empty plasmid does not cause aggregation (Fig. S5); hence, if a necessary tyrosine is mutated, there should be a reduction in aggregation. Aggregation was always observed with TpbB-Y95F in nine cultures; hence TpbB-Y95F remains active even though it lacks tyrosine 95 so this tyrosine is not phosphorylated/dephosphorylated. In contrast, the Y48F mutation of TpbB decreased aggregation for 43% of the cultures (20 of 46 cultures did not aggregate), and the Y62F mutation decreased aggregation for 24% of the cultures (9 of 37 cultures did not aggregation). Hence, both Y48 and Y62 are likely targets for tyrosine phosphorylation/dephosphorylation of TpbB with Y48 preferred. We confirmed that these mutations did not affect expression level of TpbB protein (data not shown).

### TpbA tyrosine phosphatase is unique among bacteria and eukaryotes

Tyrosine phosphorylation and dephosphorylation have crucial roles in cellular signaling and are well-conserved among many organisms [33]. Some bacterial tyrosine phosphorylations have been identified and these regulatory systems control divergent cellular functions [34]. In order to predict whether TpbA function is conserved among other species, we conducted a BLASTP search and found the TpbA protein is highly conserved among *P. aeruginosa* (PAO1, PA14, C3719, and PA7 with an E value less than 3^e-98^) and is well-conserved among *P. fluorescens* Pf-5, *P. fluorescens* Pf0-1, *P. mendocina*, *Burkholderia cepacia*, *Pelobacter carbinolicus*, *Desulfatibacillum alkenivorans*, *Bacteroides thetaiotaomicron*, *B. ovatus*, *B. caccae*, *Acinetobacter baumannii*, *Desulfococcus oleovorans*, and *Geobacter metallireducens* (E values less than 3^e-11^). All of these conserved tyrosine phosphatases have the C(X)_5_R(S/T) signature and most are uncharacterized. Even though protein similarity is not very high, some eukaryotes, such as *Homo sapiens* and *Arabidopsis thaliana*, have TpbA homologs with a C(X)_5_R(S/T) signature. Therefore, TpbA and TpbA homologs may share important functions in procaryotes and eucaryotes.

### Las-QS regulates expression of *tpbA*

**Figure 7:**
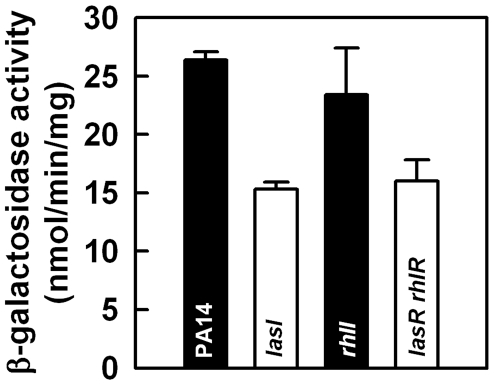
Las QS activates transcription of *tpbA*. β-galactosidase activity of p*tpbA* was measured with biofilm cells of PA14 and the mutants *lasI*, *rhlI*, and *lasR rhlR* using pLP-p*tpbA*. Data show the average of the two independent experiments±s.d.

QS regulates many genes in *P. aeruginosa* via a conserved *cis*-element in the promoter of each gene. *N*-(3-oxododecanoyl)-L-homoserine lactone binds to the LasR transcriptional regulator [35], and this complex interacts with the *las-*box, defined as CT-(N)_12_-AG sequence [36]. The Las-box is conserved among the promoters of the Las-QS regulated genes including *lasB*, *rhlAB*, and *rhlI* [36]. Another class of transcriptional regulation is governed by the *lys*-box, that is defined as a palindromic sequence, T-(N)_11_-A [37], and MvfR is a LysR-type transcription factor that binds to the *lys*-box [38]. We found that the promoter of *tpbA* (p*tpbA*) has a putative *las-*box 220 bp upstream of the start codon ( CTCGCCTCGCTGAAAG ) and a putative *lys*-box 90 bp upstream of the start codon ( TGAAGCTGCCTCA ). In order to examine if expression of *tpbA* is regulated by QS, we constructed a p*tpbA*::lacZ fusion plasmid (pLP-p*tpbA*) and transformed this into QS-related PA14 mutants (*lasI*, *rhlI*, and *lasR rhlR*). Expression of *tpbA* gene in biofilm cells was reduced by 42% in the *lasI* mutant, but not in the *rhlI* mutant (Fig. 7). Corroborating these results, inactivation of both *lasR* and *rhlR* also decreased expression of *tpbA* gene by 39% (Fig. 7). Similar results were obtained when the activity of p*tpbA*::*lacZ* was examined in planktonic cells (50% reduction in transcription for the *lasI* mutant and 37% reduction for the *lasR rhlR* mutant). Since loss of QS only affected expression of *tpbA* by 50%, other factors may also participate in the regulation of *tpbA*. These results suggest that Las-QS, rather than Rhl-QS, is an activator of *tpbA* expression with other unknown regulators.

We also investigated whether the *tpbA* mutation influences the regulation of Las- and Rhl-QS using p*lasR*::*lacZ* and p*rhlR*::*lacZ* plasmids. Expression of *lasR* was slightly increased (1.3-fold) with the *tpbA* mutation. This indicated that Las-QS has more impact on expression of *tpbA* than *tpbA* does on that of Las-QS. In addition, expression of *rhlR* was decreased by 2-fold in the *tpbA* mutant. Hence, LasR appears to enhance *tpbA* transcription and TpbA leads to increased *rhl* transcription.

## Discussion

**Figure 8:**
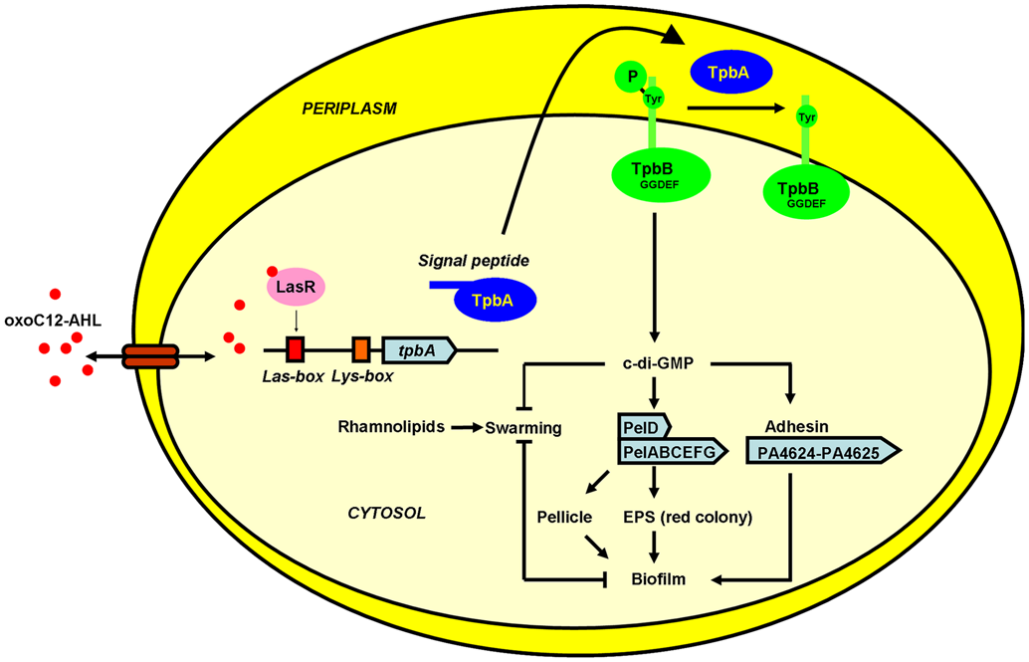
Schematic of TpbA regulation of biofilm formation in *P. aeruginosa* PA14. The QS molecule, *N*-(3-oxododecanoyl)-L-homoserine lactone (3-oxoC12-HSL), binds to the LasR transcription factor, and this complex activates expression of *tpbA*. TpbA has a N-terminal signal sequence and is translocated into the periplasm. Periplasmic TpbA dephosphorylates the membrane-anchored GGDEF protein TpbB at a tyrosine reside which deactivates GGDEF protein activity. The reduced cellular c-di-GMP concentration decreases expression of the *pel* operon as well as adhesin genes. This leads to reduced EPS production, biofilm formation, and pellicle formation, as well as enhanced swarming motility. Production of rhamnolipids is not regulated by TpbA.

In this study, we demonstrate that TpbA is a tyrosine phosphatase that regulates diverse phenotypes in *P. aeruginosa* including the concentration of cellular c-di-GMP. As a second messenger, c-di-GMP is a positive regulator of biofilm formation [4], EPS production [6], and pellicle formation [4], and a negative regulator of swarming motility [39]. The lines of evidence that show TpbA represses c-di-GMP production in *P. aeruginosa* that we found are (i) inactivating *tpbA* increases c-di-GMP (Fig. S3); (ii) inactivating *tpbA* increases biofilm formation (Fig. 1), EPS production (Fig. 3B), and pellicle formation, and c-di-GMP stimulates biofilm formation [4], EPS production [6], and pellicle formation [4]; (iii) inactivating *tpbA* increases expression of the *pel* locus (seen via the whole-transcriptome analysis and RT-PCR), and c-di-GMP activates expression of *pelA* [6]; (iv) inactivating *tpbA* increases aggregation (Fig. 4) and expression of adhesins (PA4625), and c-di-GMP stimulates adhesion [40]; (v) inactivating *tpbA* decreases motility (abolishing swarming and decreasing swimming in the *tpbA* mutant, Fig. 2AB), and c-di-GMP decreases swarming [40]; (vi) inactivating *tpbB* (encodes a GGDEF-motif protein that produces c-di-GMP [4]) suppresses the phenotypes observed in the *tpbA* mutant, and (vii) expression of *tpbA* and *tpbB* in *trans* complements aggregation/biofilm formation and aggregation, respectively. Thus, TpbA represses these phenotypes by decreasing c-di-GMP. A proposed regulatory mechanism for biofilm formation by TpbA is shown in Fig. 8.

EPS production in *P. aeruginosa* PA14 is regulated by PelA [3]. Transcription of *pelA* is higher at temperatures lower than 37°C, and PA14 forms more biofilm at lower temperatures [7]. However, the *tpbA* mutation seems to constitutively enhance *pel* expression independently from this temperature regulation as seen in the enhanced EPS production at 37°C (Fig. 3) and the whole-transcriptome analysis that was conducted at 37°C (Table S2). In addition to increased expression of *pelA*, additional activation of Pel proteins might be caused by the increased c-di-GMP concentration by the *tpbA* mutation since c-di-GMP binds PelD and increases EPS production [6]. In addition to the enhanced EPS production (Fig. 3B) and increased *pel* expression (Fig. 3A, Table S1, and qRT-PCR) seen in the *tpbA* mutant, another reason why inactivating *tpbA* increased biofilm formation is the elevated adhesin activity as seen via enhanced biofilm formation on the bottom of polystyrene plates (Fig. 1B). Cell surface adhesins affect bacterial adhesive activity [41], and we have discovered a novel adhesin (PA4625) that is related to TpbA (Table S1) and to initial biofilm formation (Fig. 4B). Since expression of adhesion factors is also positively regulated by c-di-GMP [40], elevated c-di-GMP level enhances adhesion of the *tpbA* mutant.

c-di-GMP seems to control the switch of motility-sessility of the *tpbA* mutant since inactivation of TpbA abolished swarming motility (Fig. 2A) and decreased swimming motility by 40% (Fig. 2B), although regulation of swarming motility is very complex as its activity is controlled by QS, flagellar synthesis, and production of rhamnolipids [42]. In addition, our whole-transcriptome results showed weak repression of some of flagellar biosynthesis genes (*flg*, *fle*, and *fli* loci) due to the elevated c-di-GMP, and activity of FleQ, a transcriptional activator of flagellar biosynthesis, is repressed upon binding c-di-GMP [43]. Hence, the increased c-di-GMP concentrations may repress motility of the *tpbA* mutant via the FleQ pathway that affects expression of flagellar synthesis genes.

Many genes are expected to be differentially regulated by changing c-di-GMP concentrations since it plays a role as a second messenger in *P. aeruginosa*. Similar regulation of gene expression was observed between the *tpbA* mutant and the other strains related to c-di-GMP production. For example, production of PA1107, TpbB, and PA3702 proteins that have a GGDEF-domain leads to activation of *pelA* expression [6]. Mutation in *wspF*, encoding a CheB-like methylesterase, increases both biofilm formation and c-di-GMP production [25]. *wspF* mutation altered expression of genes such as *pelABCDEFG*, PA4624, PA4625, PA2440, and PA2441 whose expression are induced in the *tpbA* mutant (Table S2). Common regulation of these genes may be partially controlled by the elevated cellular c-di-GMP concentrations. In contrast, expression of *pelA* was not induced in the *bifA* mutant that produces more c-di-GMP [44]. This may be because regulation of c-di-GMP signaling is complex in that the *P. aeruginosa* genome encodes 17 diguanylate cyclases, 5 phosphodiesterases, and 16 diguanylate cyclase-phosphodiesterase proteins [4].

Relevance of c-di-GMP to regulation of diverse cellular functions is now an emerging topic in bacteriology. This second messenger is an activator of cellulose synthase in *Acetobactor xylinum* [5] and controls many phenotypes in *P. aeruginosa* [4]. Several GGDEF proteins for synthesis and EAL proteins for degradation of c-di-GMP have been identified in *P. aeruginosa* [6],[39],[44], and increased production of c-di-GMP enhances biofilm formation and decreases swarming motility [4],[6],[39],[44]. Similarly, enhanced biofilm formation and/or abolished swarming motility were observed in the *tpbA* mutant via increased production of cellular c-di-GMP. Since TpbA does not possess GGDEF and EAL domains, this protein indirectly influences cellular c-di-GMP concentrations via its phosphatase activity as shown by activity with both pNPP, a broad substrate for phosphatases, and two phosphotyrosine-specific peptides (Fig. 6). We also found that processing the N-terminal signal sequence may be necessary for TpbA activity in the periplasm. Hence, our results reveal a novel regulatory mechanism for cellular c-di-GMP concentration by tyrosine phosphorylation in the periplasm of *P. aeruginosa*; control of c-di-GMP by tyrosine phosphorylation has not been shown previously.

There is little known about the regulation of GGDEF and EAL proteins in regard to regulation of c-di-GMP level. A chemosensory system, encoded by *wspABCDEFR* in PAO1, regulates c-di-GMP production via a His-Asp phosphorylation relay [25]. For the *tpbA* mutant, *tpbB* was found to reverse the phenotype of *tpbA*, suggesting that overproduction of c-di-GMP is clearly related to the phenotypes of the *tpbA* mutant as overexpression of this gene caused pronounced aggregation (Fig. S2). Probably, TpbB, or another GGDEF protein, might participate in c-di-GMP synthesis in the *tpbA* mutant. A comprehensive analysis of all of the *P. aeruginosa* GGDEF proteins has been completed, and those GGDEF proteins that abolished or decreased biofilm formation are PA0169, PA1107, TpbB, PA1181, PA1433, PA1727, PA3702, PA4959, and PA5487 [4]. Among these GGDEF proteins, only PA1107, TpbB, PA3702, and PA5487 increased biofilm formation when their genes were overexpressed [4]. Because TpbA is a periplasmic protein, its target GGDEF protein should have periplasmic regions. By a bioinformatics evaluation, of those four GGDEF proteins that increased biofilm formation, only PA1107 and TpbB have transmembrane regions. Taken together with the results of genetic screening, TpbB is the most likely target protein for TpbA. Also, our results imply the periplasmic Y48 and Y62 residues of TpbB are the likely targets for tyrosine phosphorylation. We are now investigating whether TpbA regulates the activity of GGDEF proteins to control cellular c-di-GMP concentrations in *P. aeruginosa*.

The relationship between tyrosine phosphorylation and biofilm formation is not well established. We found that trisodium orthovanadate treatment increased biofilm formation of PA14 (Fig. S4), indicating that tyrosine phosphorylation increases biofilm formation in *P. aeruginosa*. Recently, Ltp1, a low molecular weight tyrosine phosphatase in non-motile, Gram-negative *P. gingivalis*, was identified as a negative regulator of EPS production and biofilm formation [45]. A sequence similarity search shows TpbA is not a homolog of Ltp1, because TpbA has a signal sequence in its N-terminal region, and TpbA is translocated into the periplasm. Other differences were found in the position of the motif for the tyrosine phosphatase, since TpbA has the motif at the position 132 and Lpt1 has it at position 9. It appears the *P. aeruginosa* genome encodes another tyrosine phosphatase, annotated as *ptpA*, that has a tyrosine phosphatase motif at the position 7 and does not have a signal sequence at the N-terminus. The function of PtpA is unknown but it is essential [46].

In contrast to poorly-investigated Tyr phosphorylation, regulation of biofilm formation by phosphorylation has been identified for several systems; for example, for the His kinase/Asp response regulator phosphorylation systems RocS1/RocA1/RocR of *P. aeruginosa* PAK [47] and the PAO1 PA1611/PA1976/PA2824/RetS/HptB system of *P. aeruginosa* [48]. In addition, in the *B. subtilis* PrkC/PrpC system [18], loss of a membrane-anchored Thr kinase and its phosphatase reduces biofilm formation. Our results indicate that TpbA acts as a negative regulator of cellular c-di-GMP formation and loss of TpbA results in increased c-di-GMP concentrations that enhance biofilm formation and inhibit motility. These results show clearly that posttranslational modification through phosphatase activity is related to bacterial biofilm formation as well as to the regulation of the synthesis of cellular second messengers. In addition, by showing *tpbA* transcription is increased by LasR (Fig. 7) and by finding AHL-binding motifs, we have now linked quorum sensing to c-di-GMP concentrations and biofilm formation in *P. aeruginosa*. Similarly, *Vibrio cholerae* QS was found recently to reduce cellular c-di-GMP concentrations via a c-di-GMP-specific phosphodiesterase which leads to lower biofilm formation [49]. A common element in both studies is that QS seems to be a negative regulator of c-di-GMP. The *tpbA* mutation caused a hyper-aggregative phenotype (Fig. 4), and this would lead to flat biofilms since the *wspF* mutant, which accumulates increased c-di-GMP, formed flat biofilms [25]. Formation of flat and undifferentiated biofilms is also observed by loss of LasI function [12] that can activate *tpbA* expression. Hence, TpbA might participate in developing biofilm structure. These results are important in that the regulatory networks that control c-di-GMP concentrations are now linked to the environment and cell populations.

## Materials and Methods

### Bacterial strains and growth conditions

Strains used in this study are listed in Table 2. *P. aeruginosa* PA14 wild-type and its isogenic mutants were obtained from the Harvard Medical School [46]. Transposon insertion of the *tpbA* mutant was verified as described previously with a minor modification [50]. Briefly, the *tpbA* gene was amplified from chromosomal DNA using primers PA14_13660-VF and PA14_13660-VR (Table S3) which did not amplify chromosomal DNA from the *tpbA* mutant. In addition, the DNA fragment corresponding to the end of the transposon and *tpbA* gene was amplified with *tpbA* chromosomal DNA using primers PA14_13660-VF and GB-3a (Table S3) and PA14_13660-VR and R1 (Table S3) but these pairs of primers did not amplify PA14 wild-type chromosomal DNA. *P. aeruginosa* and *E. coli* were routinely grown in Luria-Bertani (LB) medium at 37°C unless noted. Gentamicin (15 µg/mL) and tetracycline (75 µg/mL) were used for growth of the *P. aeruginosa* transposon mutants, carbenicillin (300 µg/mL) was used to maintain *P. aeruginosa* plasmids, and kanamycin (50 µg/mL) and chloramphenicol (50 µg/mL) were used to maintain *E. coli* plasmids (Table 2).

**Table 2:** Strains used in this study.

Strain	Genotype or description	Reference
***P. aeruginosa***		
PA14	Wild-type strain	[46]
PA14_13660 (PA3885, *tpbA*)	PA14_13660 Ω *Mar2xT7*, Gm^R^	[46]
PA14_19120 (PA3477, *rhlR*)	PA14_19120 Ω *Mar2xT7*, Gm^R^	[46]
PA14_50360 (PA1086, *flgK*)	PA14_50360 Ω *Mar2xT7*, Gm^R^	[46]
PA14_49880 (PA1121)	PA14_49880 Ω *Mar2xT7*, Gm^R^	[46]
PA14_49890 (PA1120, *tpbB*)	PA14_49890 Ω *Mar2xT7*, Gm^R^	[46]
PA14_42820 (PA1678)	PA14_42820 Ω *Mar2xT7*, Gm^R^	[46]
PA14_67780 (PA5132)	PA14_67780 Ω *Mar2xT7*, Gm^R^	[46]
PA14_24480 (PA3064, *pelA*)	PA14_24480 Ω *Mar2xT7*, Gm^R^	[46]
PA14_24490 (PA3063, *pelB*)	PA14_24490 Ω *Mar2xT7*, Gm^R^	[46]
PA14_24510 (PA3061, *pelD*)	PA14_24510 Ω *Mar2xT7*, Gm^R^	[46]
PA14_24560 (PA3058, *pelG*)	PA14_24560 Ω *Mar2xT7*, Gm^R^	[46]
PA14_61190 (PA4624)	PA14_61190 Ω *Mar2xT7*, Gm^R^	[46]
PA14_61200 (PA4625)	PA14_61200 Ω *Mar2xT7*, Gm^R^	[46]
PA14_45940 (PA1432, *lasI*)	PA14_45940 Ω *Mar2xT7*, Gm^R^	[46]
PA14_19130 (PA3476, *rhlI*)	PA14_19130 Ω *Mar2xT7*, Gm^R^	[46]
PA14_lasR rhlR (PA1431 PA3477, *lasR rhlR*)	PA14 *ΔlasR ΔrhlR*	[70]
***E. coli***		
BL21(DE3)	F^−^ *ompT hsdS_B_(r_B_^−^m_B_^−^) gal dcm λ*(DE3) Ω p*lac*UV5:: T7 polymerase	Novagen
HB101	*pro leu thi lacY* Str*^r^ endoI^−^ recA^−^ r^−^ m^−^*	[67]
S17-1(*λpir*)/pUT-miniTn*5*-*luxAB*	Tc^R^ Sm^R^ Tp^R^ *mod* ^+^ *res thi pro recA hsdR17* Ω RP4-TC::Mu-Km::Tn7 with pUT-miniTn*5-luxAB*	[59],[71]
TG1	K12, *lac–pro supE thi hsdD5* (F′ *traD36 proA* ^+^ *B* ^+^ *lacI* ^q^ *lacZ* M15)	[61]
AG1	*recA1 endA1 gyrA96 thi-1 hsdR17(r_K_^−^ m_K_^−^) supE44 relA1*	[72]
**Plasmids**		
pMQ70	Car^R^, Ap^R^, P_BAD_, expression vector	[51]
pMQ70-*tpbA*	Car^R^, Ap^R^, P_BAD_::*tpbA*, complementation plasmid	This study
pMQ70-*tpbB*	Car^R^, Ap^R^, P_BAD_::*tpbB*, complementation plasmid	This study
pMQ70-*tpbB-*Y48F	Car^R^, Ap^R^, P_BAD_::*tpbB*, Y48 of TpbB replaced with F48	This study
pMQ70-*tpbB-*Y62F	Car^R^, Ap^R^, P_BAD_::*tpbB*, Y62 of TpbB replaced with F62	This study
pMQ70-*tpbB-*Y95F	Car^R^, Ap^R^, P_BAD_::*tpbB*, Y95 of TpbB replaced with F95	This study
pRK2013	Mobilizing conjugation plasmid	[68]
pET28b	Km^R^, P_T7_ expression vector	Novagen
pET28b-13660n	Km^R^, P_T7_::*tpbA-nHis* ^+^, expression vector for TpbA-nHis	This study
pET28b-13660c	Km^R^, P_T7_::*tpbA-cHis* ^+^, expression vector for TpbA-cHis	This study
pLP170	Car^R^ Ap^R^, promoterless-lacZ	[73]
pLP-p*tpbA*	Car^R^ Ap^R^, p*tpbA*::lacZ	This study
pPCS1001	Car^R^ Ap^R^, p*lasR*::lacZ	[73]
pPCS1002	Car^R^ Ap^R^, p*rhlR*::lacZ	[73]
pCA24N	Cm^R^, *lacI* ^q^, pCA24N	[72]
pCA24N-*yddV*	Cm^R^, *lacI* ^q^, pCA24N P_T5-lac_::*yddV* ^+^	[72]
pCA24N-*cpdB*	Cm^R^, *lacI* ^q^, pCA24N P_T5-lac_::*cpdB* ^+^	[72]
pCA24N-*oxyR*	Cm^R^, *lacI* ^q^, pCA24N P_T5-lac_::*oxyR* ^+^	[72]
pGEM-T easy	Car^R^ Ap^R^, TA cloning vector	Qiagen

Gm^R^, Tc^R^, Km^R^, Car^R^, Cm^R^, and Ap^R^ indicate gentamicin, tetracycline, kanamycin, carbenicillin, chloramphenicol, and ampicillin resistance, respectively.

### Complementation of *P. aeruginosa* mutants

For complementation of the *tpbA* and *tpbB* mutations, *tpbA* and *tpbB* were expressed under the control of the pBAD promoter in pMQ70 [51]. *tpbA* and *tpbB* were amplified using a Pfu DNA polymerase with primers PA14_13660-F1-NheI and PA14_13660-R-cHis-HindIII and PA14_49890-F1-NheI and PA14_49890-R-cHis-HindIII, respectively (Table S3). PCR products were cloned into the NheI and HindIII sites of pMQ70. The resulting plasmids, pMQ70-*tpbA* and pMQ70-*tpbB*, were transformed into PA14 and the mutants by conjugation. Briefly, 1 mL of overnight culture of the recipient strain (PA14 or the mutant), helper strain (HB101/pRK2013), and donor strain (TG1/pMQ70, TG1/pMQ70-*tpbA*, or pMQ70-*tpbB*) was washed with 1 mL of fresh LB medium. The mixture of three strains was incubated on LB plates at 37°C overnight. PA14 strains with pMQ70-based plasmid were selected on LB plates with 100 µg/mL rifampicin (to kill the donor and helper), 300 µg/mL carbenicillin (to kill *P. aeruginosa* without pMQ70-based plasmids), and 15 µg/mL gentamicin (if a recipient was a PA14 mutant constructed using a transposon insertion with the Gm^R^ gene). If indicated, 0.05% arabinose was added to induce gene expression.

### Biofilm formation

Biofilm formation was examined in 96-well polystyrene plates using crystal violet staining [52]. Overnight cultures of *P. aeruginosa* were diluted to a turbidity of 0.05 at 600 nm with fresh LB medium, and then 150 µL of diluted bacterial culture was incubated in 96-well polystyrene plates for 2, 4, 8, 24, and 50 h. Ten wells were used for each strain and three independent cultures were used for each experiment. Trisodium orthovanadate, a tyrosine phosphatase-specific inhibitor, was added to LB medium at 10 mM.

### Colony morphology

To observe colony morphology, overnight cultures were diluted to a turbidity of 0.005 at 600 nm with T-broth (10 g/L tryptone), and 2 µL of diluted cultures were spotted on Congo-red plates (10 g/L tryptone, 40 µg/mL Congo-red, and 20 µg/mL Coomassie brilliant blue) [3]. Plates were incubated at 37°C or room temperature for 3 to 7 days.

### Motility assays

Swimming motility was examined with cells grown to a turbidity of 1 at 600 nm using 0.3% agar plates with 1% tryptone and 0.25% NaCl [53] and swarming motility was examined with BM-2 plates (62 mM potassium phosphate, 2 mM MgSO_4_, 10 µM FeSO_4_, 0.1% casamino acid, 0.4% glucose, and 0.5% Bacto agar) [54]. Motility was measured after 24 h. Five plates were tested for each culture, and two independent cultures were used. The *flgK* [22] and *rhlR* [23] mutants were used as negative controls for swimming and swarming, respectively.

### Aggregation assay

Aggregation was examined by diluting overnight cultures with fresh LB medium in 5 mL screw-capped tubes from 0% (no added fresh LB medium) to 100% (pure fresh LB medium). Cells were inverted gently several times and placed at room temperature for 15 min.

### Pellicle formation

Overnight cultures of PA14, the *tpbA* mutant, and the *pelA* mutant were diluted to a turbidity of 0.005 at 600 nm in 4 mL T-broth, and the bacterial cultures were placed in a polycarbonate glass tube at 37°C or room temperature [3].

### EPS assay

Pel-dependent EPS production was quantified as described previously [6] based on the amount of Congo red that binds to the EPS. Briefly, 1 mL of overnight culture was washed with 1 mL T-broth. Due to aggregative phenotype of the *tpbA* mutant, cell pellets of the *tpbA* mutant, wild-type, and *pelA* mutant (negative control) were sonicated three times at 3W for 10 sec. Bacterial suspensions in T-broth (500 µL) were incubated with 40 µg/mL Congo-red at 37°C or room temperature with vigorous shaking. After 2 h, the absorbance of the supernatants of the each suspension was measured at 490 nm using a spectrophotometer. T-broth with 40 µg/mL Congo-red was used as a blank.

### Rhamnolipids assay

Production of rhamnolipids was determined as described previously [55]. Overnight cultures were diluted to a turbidity of 0.05 at 600 nm in 25 mL LB medium and were re-grown at 250 rpm for 24 h to eliminate the effect of antibiotics. The supernatants of the bacterial cultures were used to determine the relative concentrations of rhamnolipids using orcinol/sulfuric acid. Rhamnose (Fisher Scientific, Pittsburgh, PA) was used as a standard.

### Whole-transcriptome analysis

The *P. aeruginosa* genome array (Affymetrix, P/N 510596) was used to investigate differential gene expression in biofilm cells between PA14 and the *tpbA* mutant. Biofilm cells were harvested from 10 g of glass wool [56] after incubation for 4 h and 7 h in LB with shaking at 250 rpm, and RNA was extracted with a RNeasy Mini Kit (Qiagen) [57]; note the RNase inhibitor RNA*later* (Applied Biosystems, Austin, TX) was used for the 4 h and second 7 h set of microarrays. Global scaling was applied so the average signal intensity was 500. The probe array images were inspected for any image artifact. Background values, noise values, and scaling factors of both arrays were examined and were comparable. The intensities of polyadenosine RNA controls were used to monitor the labeling process. If the gene with the larger transcription rate did not have a consistent transcription rate based on the 13 probe pairs (*p*-value less than 0.05), these genes were discarded. A gene was considered differentially expressed when the *p*-value for comparing two chips was lower than 0.05 (to assure that the change in gene expression was statistically significant and that false positives arise less than 5%) and when the expression ratio was higher than the standard deviation for the whole microarrays [58], 1.4 for 4 h, 1.7 for the first 7 h replicate, and 2.2 for second 7 h replicate. All three sets of whole-transcriptome data were deposited at the Gene Expression Omnibus (GSE13871).

### qRT-PCR

qRT-PCR was performed using the StepOnePlus™ Real-Time PCR System (Applied Biosystems, Foster City, CA). Expression of *pelA*, the PA4625 gene, and the PA4139 gene was determined using total RNA isolated from two independent biofilm cultures of PA14 and the *tpbA* mutant. The biofilm cells were grown and total RNA were isolated in the same manner as described above for the whole-transcriptome analysis. The primers for qRT-PCR are listed in Table S3. The housekeeping gene *rplU* [44] was used to normalize the gene expression data.

### Genetic screening

To isolate the suppressive loci for TpbA functions, a double mutant library was generated using the Tn*5-luxAB* transposon with the background of the *tpbA* mutation as described previously [59]. Briefly, 1 mL of overnight culture of the *P. aeruginosa tpbA* mutant and *E. coli* S17-1 (λpir) with Tn*5*-*luxAB* were grown on LB plates together overnight. Cells were harvested from the plate and resuspended in 10 mL of LB medium. Screening of cells with mutations in addition to *tpbA* was performed in three steps. Suppression of the highly-aggregative phenotype of the *tpbA* mutant was used first; the cell mixture (*P. aeruginosa* single and double mutants along with *E. coli* S17-1) was placed at room temperature for 15 min and the supernatant was used for secondary screening (cells with the *tpbA* mutant aggregative phenotype were therefore discarded). Supernatant cells were spread on Congo-red plates with 50 µg/mL gentamicin (to kill *E. coli*), and 75 µg/mL tetracycline (to kill the *tpbA* single mutant), and incubated for 3–4 days. *P. aeruginosa* double mutants with smooth surfaces were picked (the *tpbA* mutant was red and wrinkled). The crystal violet biofilm assay was used for the third screening, and mutants showing decreased biofilm formation in comparison to that of the *tpbA* mutant were chosen as phenotype reversal mutants. The insertion position of Tn*5-luxAB* transposon was determined by two-step PCR as described previously [59] with primers LuxAB inside and Arb1 for the first round of PCR and LuxAB outside and Arb2 for the second round of PCR (Table S3). The PCR product was ligated into pGEM-T easy (Promega, Madison, MI) and sequenced using a BigDye Terminator Cycle Sequencing Kit (Applied Biosystems, Foster City, CA).

### Quantification of c-di-GMP

c-di-GMP was isolated as described previously [60]. *P. aeruginosa* was grown in 1 L of LB medium for 16 h at 250 rpm, and formaldehyde (final concentration of 0.18%) was added to inactivate degradation of c-di-GMP. Cells were harvested by centrifugation at 8,000 *g* for 10 min at 4°C. Nucleotide extract was prepared as described previously [60]. Cell pellets were washed with 40 mL of phosphate buffered saline (pH 7) [61] with 0.18% formaldehyde and centrifuged at 8,000 *g* for 10 min at 4°C. The cell pellets were dissolved in H_2_O and boiled for 10 min. After cooling the samples on ice for 10 min, nucleotides were extracted in 65% ethanol. Supernatants were transferred, and the extraction was repeated. Pooled supernatants were lyophilized, and pellets were dissolved in 1 mL of 0.15 M triethyl ammonium acetate (TEAA, pH 5.0). The samples were filtered using a PVDF filter (0.22 µm), and 20 µL of each sample was fractionated using HPLC (Waters 515 with photodiode array detector, Milford, MA) with a reverse-phase column (Nova-Pak® C18 column; Waters, 150×3.9 cm, 4 µm). Separations were conducted in 0.15 M TEAA at a 1 mL/min flow rate using gradient elution with acetonitrile (0% to 15% concentration). Synthetic c-di-GMP (BIOLOG Life Science Institute, Bremen, Germany) was used as a standard. The peak corresponding to c-di-GMP from the extract of the *tpbA* mutant was verified by co-elution with standard c-di-GMP. *E. coli* AG1/pCA24N-*yddV* that has an elevated c-di-GMP concentration [62] was also used as a positive control.

### Plasmid construction of pET28b-13660c and purification of recombinant TpbA-cHis

To determine if TpbA is a phosphatase, *tpbA* was amplified with a Pfu DNA polymerase using primers PA14_13660-F-XbaI and PA14_13660-R-XhoI (Table S3). The PCR product was digested with XbaI and XhoI and was ligated in-frame to the polyhistidine tag sequence of the pET28b vector. The resulting plasmid, pET28b-13660c has the *tpbA* gene fused to a 6× His tag at the C-terminus (TpbA-cHis) and under control of the T7 promoter. The pET28b-13660c plasmid was confirmed by DNA sequencing with the T7 promoter and T7 terminator primers (Table S3). Production of TpbA-cHis was induced in *E. coli* BL21(DE3) cells with 1 mM IPTG at room temperature overnight. TpbA-cHis was purified using a Ni-NTA resin (Qiagen, Valencia, CA) as described in a manufacturer's protocol. Purified TpbA-cHis was dialyzed against buffer (50 mM Tris-HCl, 100 mM NaCl, 10% glycerol, 0.01% Triton X-100, pH 7.5) at 4°C overnight.

### Phosphatase assay

The *p*-nitrophenyl phosphate assay (pNPP) was used to examine TpbA-cHis phosphatase activity [63]. Purified TpbA-cHis protein was incubated in 100 µL of reaction buffer (50 mM Tris-acetate, 10 mM MgCl_2_, 10 mM pNPP, 5 mM DTT, pH 5.5) at 37°C for 1 h. The reaction was quenched by adding 900 µL of 1 M NaOH. Trisodium orthovanadate, a specific inhibitor for tyrosine phosphatase [30], was used at 10 mM. *p*-nitrophenol was measured at an absorbance of 405 nm. An extinction coefficient of 1.78×10^4^ M^−1^ cm^−1^ was used to calculate the concentration of *p*-nitrophenol.

To examine if TpbA is a tyrosine specific phosphatase, a tyrosine phosphatase assay was performed using the Tyrosine Phosphatase Assay System (Qiagen). Eight micrograms of TpbA-cHis were incubated with either 50 µM phosphotyrosine peptide type I (END(pY)INASL) or peptide type II (DADE(pY)LIPQQG) in a reaction buffer (50 mM Tris-Acetate, 10 mM MgCl_2_, pH 5.5) at 37°C for 3 h. The reaction was quenched using a molybdate dye solution and incubated for 30 min at room temperature. Released phosphate was quantified by measuring the absorbance at 630 nm.

### Determination of the subcellular localization of TpbA

TpbA-cHis protein was expressed in BL21(DE3) cells with 1 mM IPTG for 4 h at 37°C. Periplasmic proteins were purified using a PeriPreps Periplasting Kit (Epicentre Technologies, Madison, WI) as well as cytoplasmic and membrane proteins. *Escherichia coli* CpdB [64] was used as a control of periplasmic protein and the *E. coli* OxyR [65] was used for the cytoplasmic control. Fractionated proteins as well as TpbB were analyzed by 12% SDS-PAGE.

### Site-directed mutagenesis of the TpbB periplasmic tyrosine residues

Site-directed mutagenesis of the predicted periplasmic tyrosine residues of TpbB was performed to convert them to phenylalanine (Y48F, Y62F, and Y95F); it was reasoned that phenylalanine would provide a similar bulky side chain but remove the hydroxyl moiety needed for phosphorylation [66]. The mutations were introduced into pMQ70-*tpbB* using Pfu DNA polymerase and QuikChange Site-Directed Mutagenesis Kit (Stratagene, La Jolla, CA), and the primers are listed in Table S3. The resulting plasmids, pMQ70-*tpbB*-Y48F, pMQ70-*tpbB*-Y62F, and pMQ70-*tpbB*-Y95F were transformed into the *tpbB* mutant by conjugation and aggregation was assayed. DNA sequencing was used to confirm the tyrosine mutations and that no other mutations were introduced into the promoter or protein-coding sequences.

### β-galactosidase activity

The promoter region of *tpbA* (p*tpbA*), including 399 bp upstream of the start codon and 31 bp of the open reading frame, was amplified using Pfu DNA polymerase with primers pPA14_13660-F-HindIII and pPA14_13660-R-BamHI (Table S3). The PCR product (430 bp) was cloned into the HindIII/BamHI sites of pLP170 to produce pLP-p*tpbA*, and it was conjugated into PA14 and the QS-related mutants using helper strain HB101/pRK2013 [67],[68]. Transformants were grown overnight in LB medium with 300 µg/mL carbenicillin, reinoculated at a turbidity of 0.05 at 600 nm, and grown for another 6 h. Biofilm cells were harvested from 4 g of glass wool after incubation for 6 h in LB at 37°C with shaking at 250 rpm. β-galactosidase activity was measured using suspension cells and biofilm cells as described previously [69]. Similarly, β-galactosidase activity of p*lasR*::*lacZ* (pPCS1001) and p*rhlR*::*lacZ* (pPCS1002) was examined in PA14 and the *tpbA* mutant.

## Supporting Information

Figure S1 Complementation of the *tpbA* mutant using biofilm formation and aggregation. Total biofilm formation (A) and bottom biofilm formation on polystyrene plates (B) by *P. aeruginosa* PA14 and the *tpbA* mutant with either pMQ70 or pMQ70-*tpbA* in LB with 300 µg/mL carbenicillin and 0.05% arabinose after 24 h at 37°C. Six wells were used for each culture. Data show the average of the two to four independent experiments±s.d. Cell aggregation of PA14 and the *tpbA* mutant with either pMQ70 or pMQ70-*tpbA* (C). Overnight cultures (1 mL) were mixed with 3 mL of fresh LB medium, and the tubes were placed at room temperature for 15 min.
(2.54 MB TIF)

Figure S2 Complementation of the *tpbB* mutant using aggregation. Bacterial cultures were grown at 37°C at 250 rpm overnight.
(6.07 MB TIF)

Figure S3 Quantification of cellular c-di-GMP concentrations by HPLC from 30 mg of cells. 10 µM synthetic c-di-GMP (A), nucleotide extract from PA14 (B), nucleotide extract from the *tpbA* mutant (C), and nucleotide extract from the *tpbA* mutant spiked with 10 µM c-di-GMP (D). Spectra of synthetic c-di-GMP (E) and nucleotide extract from the *tpbA* mutant (F).
(1.71 MB TIF)

Figure S4 Normalized biofilm formation of PA14 at 37°C in LB after 24 h with and without tyrosine phosphatase inhibitor Na_3_VO_4_ (10 mM). Six wells were used for each culture. Data show the average of the two independent experiments±s.d.
(0.49 MB TIF)

Figure S5 Aggregation is reduced by site-directed mutagenesis at Y48 and Y62 of TpbB. The *tpbB* mutant was grown in 25 mL of LB medium supplemented with carbenicillin (300 µg/mL) and gentamicin (15 µg/mL) with pMQ70 (negative control) and pMQ70-*tpbB* (positive control) (A), pMQ70-*tpbB*-Y48F (B), pMQ70-*tpbB*-Y62F (C) and pMQ70-*tpbB*-Y95F (data not shown). Percentage of cultures with each phenotype (aggregation or no aggregation are indicated). A total of 46 and 37 independent cultures was tested for *tpbB*/pMQ70-*tpbB*-Y48F and *tpbB*/pMQ70-*tpbB*-Y62F, respectively, and representative images are shown. Note that aggregates were always formed with *tpbB*/pMQ70-*tpbB* (positive control) and *tpbB*/pMQ70-*tpbB*-Y95F, but not with *tpbB*/pMQ70 (negative control).
(9.95 MB TIF)

Table S1 Partial list of induced genes in biofilm cells in LB medium after 4 and 7 h at 37°C for the *tpbA* mutant versus wild-type PA14 using three sets of DNA microarrays (note RNA*later* was used for the 4 h set and the second set at 7 h).
(0.07 MB DOC)

Table S2 Partial list of repressed genes in biofilm cells in LB medium after 4 and 7 h at 37°C for the *tpbA* mutant versus wild-type PA14 using three sets of DNA microarrays (note RNA*later* was used for the 4 h set and the second set at 7 h).
(0.10 MB DOC)

Table S3 Primers used in this study.
(0.05 MB DOC)
