# Supplementary figures and images for: Connecting Quorum Sensing, c-di-GMP, Pel Polysaccharide, and Biofilm Formation in Pseudomonas aeruginosa through Tyrosine Phosphatase TpbA (PA3885)
Source: PLoS Pathog. 2009 Jun 19;5(6):e1000483. doi: 10.1371/journal.ppat.1000483 (PMC2691606; doi:10.1371/journal.ppat.1000483)

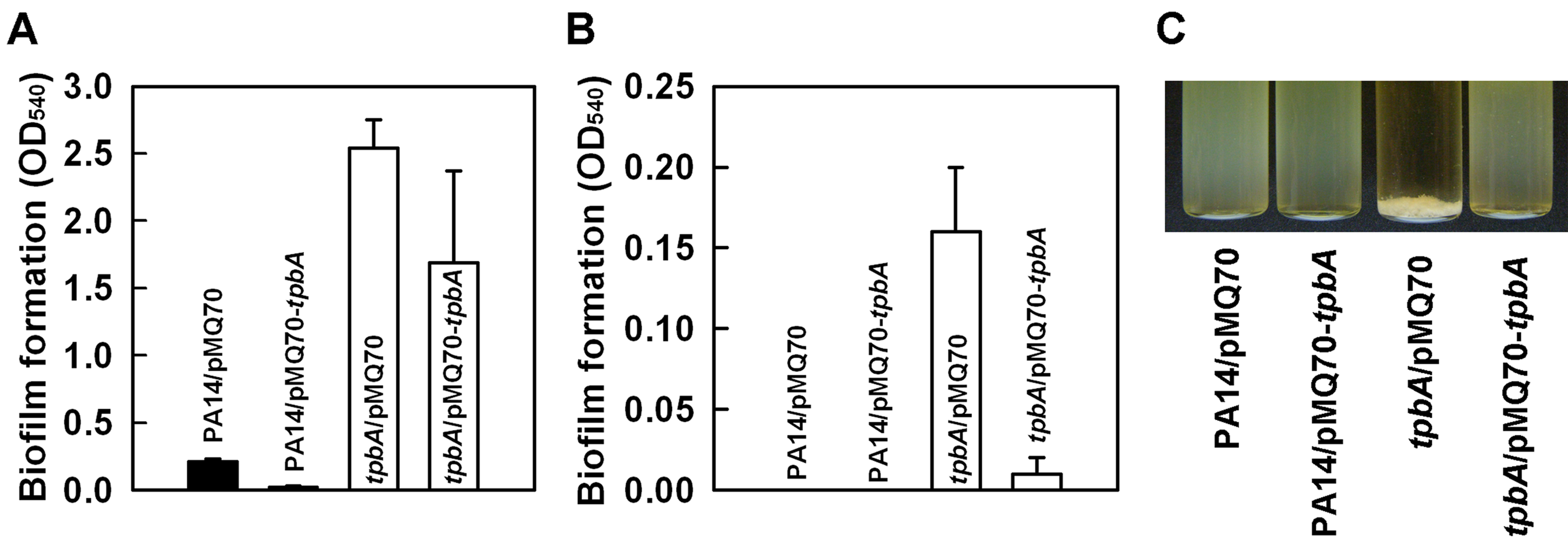

Supplement: Figure S1 — Complementation of the tpbA mutant using biofilm formation and aggregation. Total biofilm formation (A) and bottom biofilm formation on polystyrene plates (B) by P. aeruginosa PA14 and the tpbA mutant with either pMQ70 or pMQ70-tpbA in LB with 300 µg/mL carbenicillin and 0.05% arabinose after 24 h at 37°C. Six wells were used for each culture. Data show the average of the two to four independent experiments±s.d. Cell aggregation of PA14 and the tpbA mutant with either pMQ70 or pMQ70-tpbA (C). Overnight cultures (1 mL) were mixed with 3 mL of fresh LB medium, and the tubes were placed at room temperature for 15 min. (2.54 MB TIF) [file ppat.1000483.s001.tif]

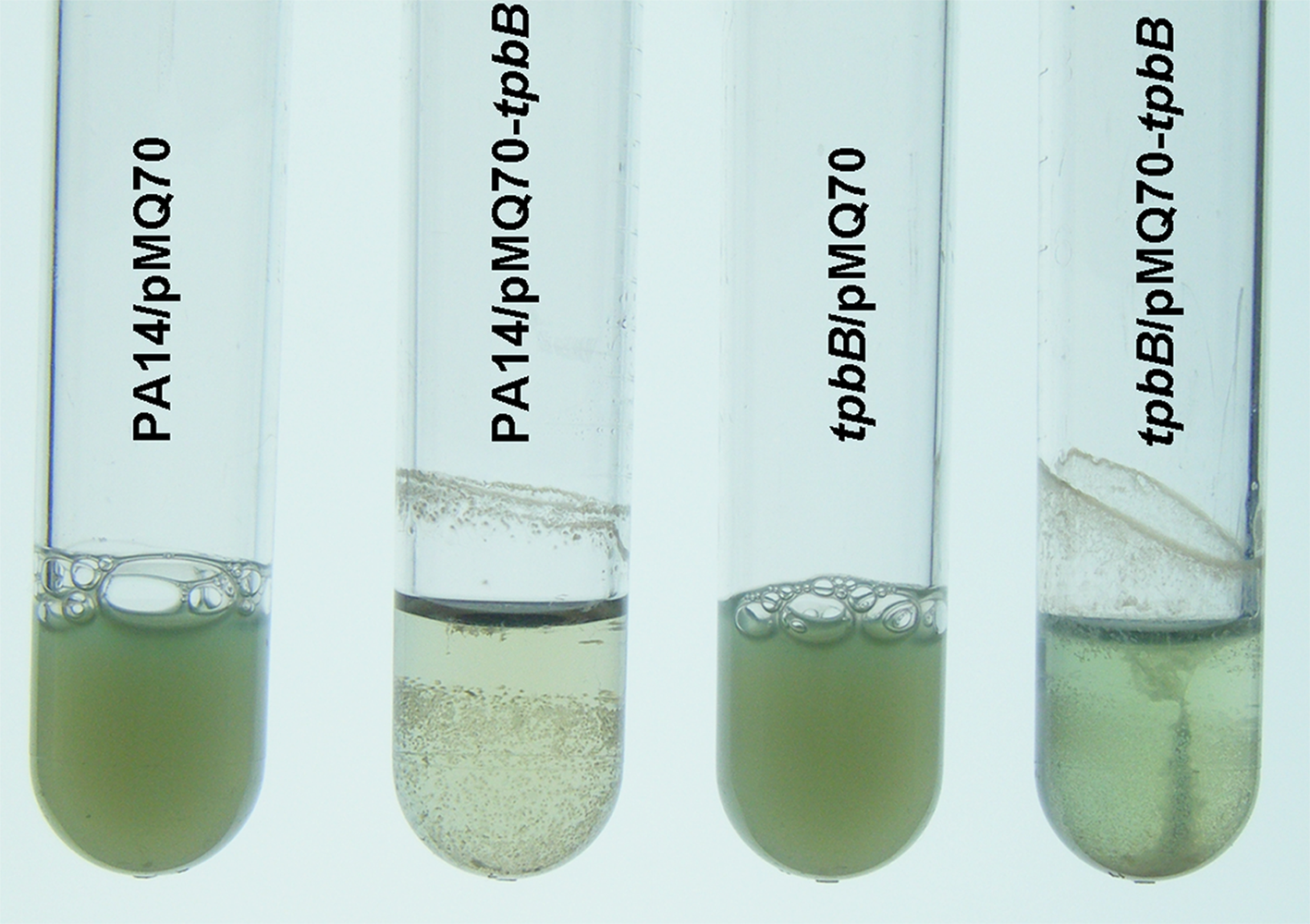

Supplement: Figure S2 — Complementation of the tpbB mutant using aggregation. Bacterial cultures were grown at 37°C at 250 rpm overnight. (6.07 MB TIF) [file ppat.1000483.s002.tif]

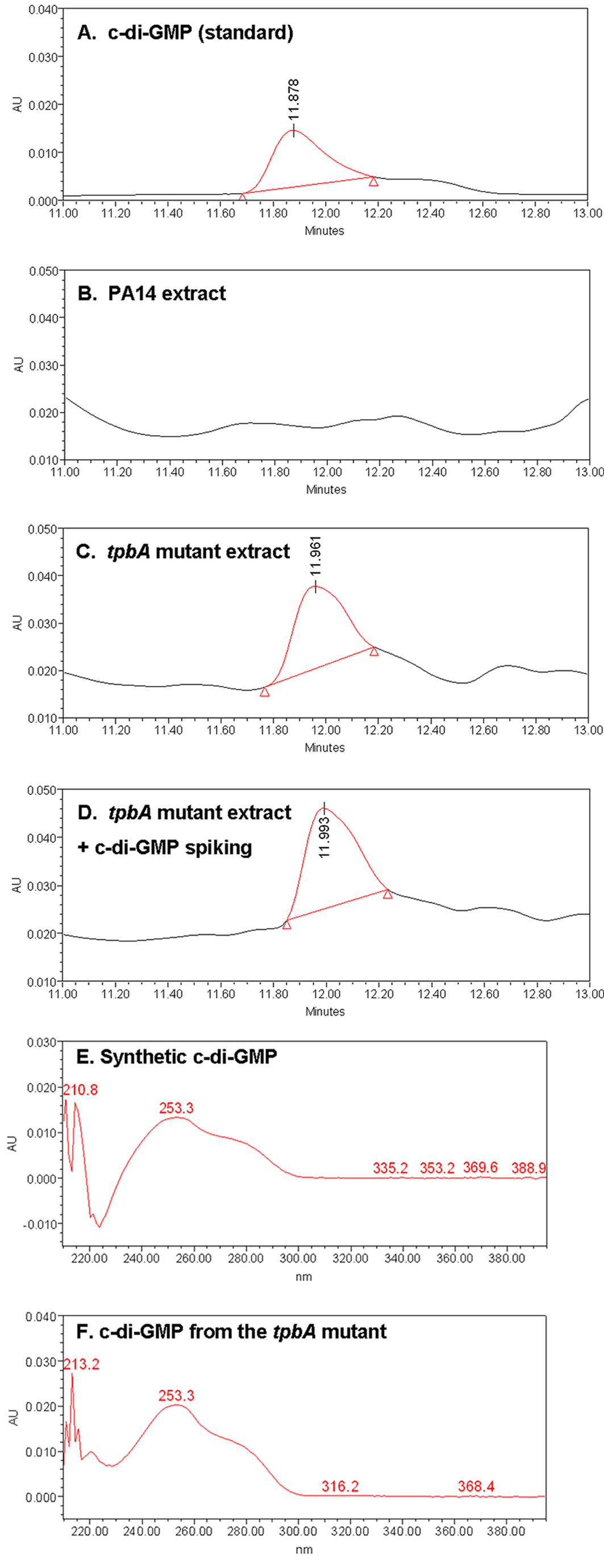

Supplement: Figure S3 — Quantification of cellular c-di-GMP concentrations by HPLC from 30 mg of cells. 10 µM synthetic c-di-GMP (A), nucleotide extract from PA14 (B), nucleotide extract from the tpbA mutant (C), and nucleotide extract from the tpbA mutant spiked with 10 µM c-di-GMP (D). Spectra of synthetic c-di-GMP (E) and nucleotide extract from the tpbA mutant (F). (1.71 MB TIF) [file ppat.1000483.s003.tif]

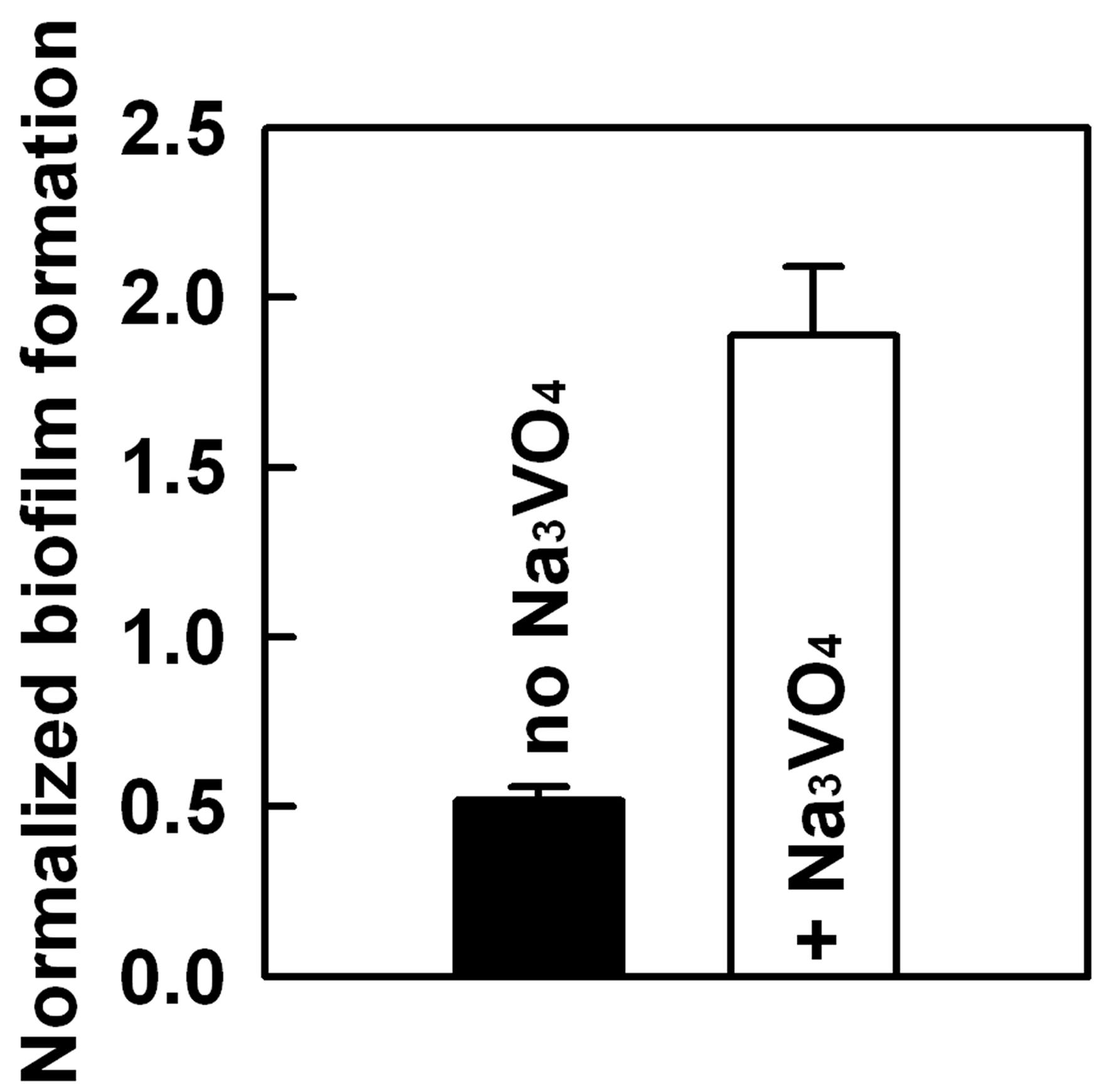

Supplement: Figure S4 — Normalized biofilm formation of PA14 at 37°C in LB after 24 h with and without tyrosine phosphatase inhibitor Na3VO4 (10 mM). Six wells were used for each culture. Data show the average of the two independent experiments±s.d. (0.49 MB TIF) [file ppat.1000483.s004.tif]

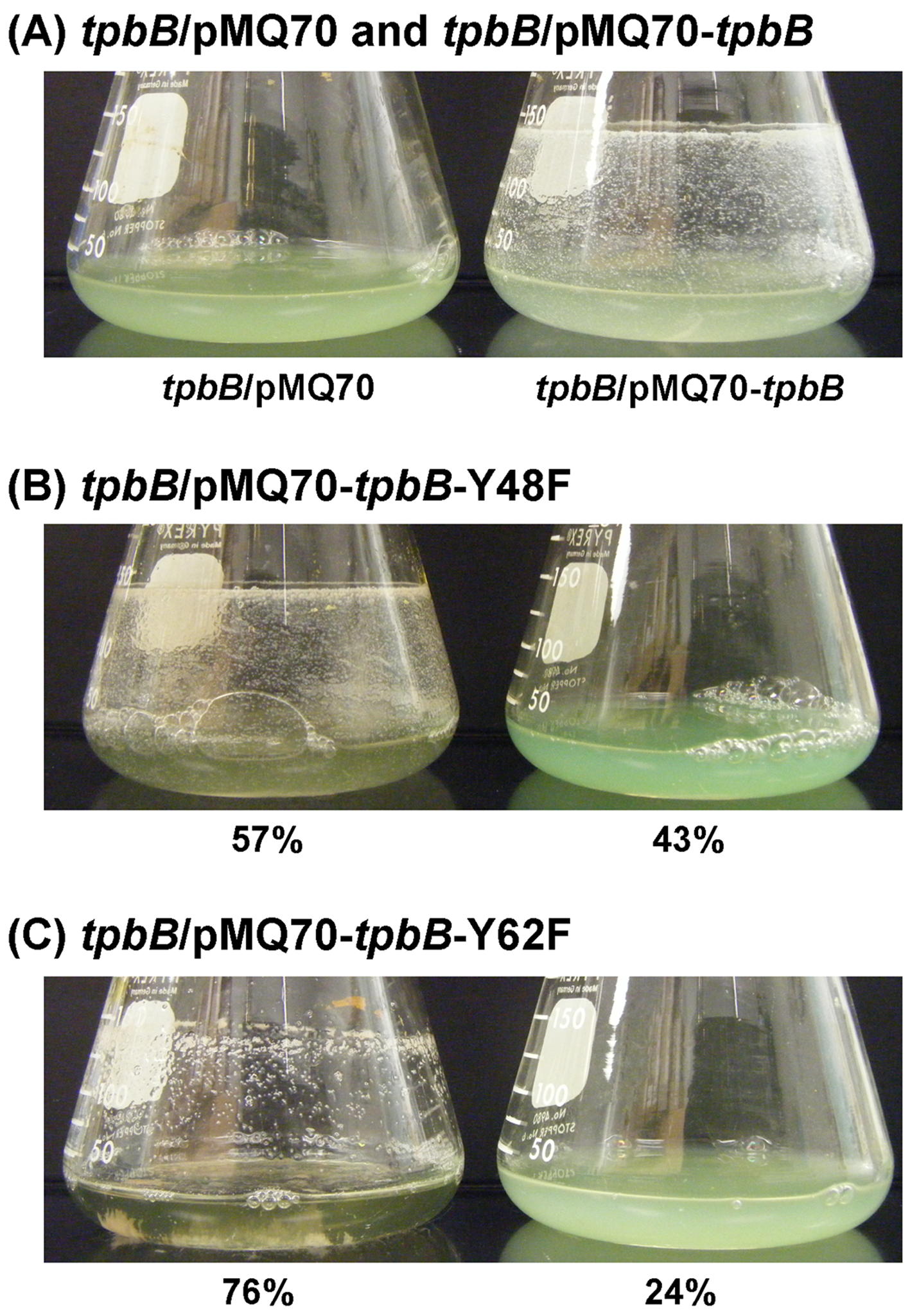

Supplement: Figure S5 — Aggregation is reduced by site-directed mutagenesis at Y48 and Y62 of TpbB. The tpbB mutant was grown in 25 mL of LB medium supplemented with carbenicillin (300 µg/mL) and gentamicin (15 µg/mL) with pMQ70 (negative control) and pMQ70-tpbB (positive control) (A), pMQ70-tpbB-Y48F (B), pMQ70-tpbB-Y62F (C) and pMQ70-tpbB-Y95F (data not shown). Percentage of cultures with each phenotype (aggregation or no aggregation are indicated). A total of 46 and 37 independent cultures was tested for tpbB/pMQ70-tpbB-Y48F and tpbB/pMQ70-tpbB-Y62F, respectively, and representative images are shown. Note that aggregates were always formed with tpbB/pMQ70-tpbB (positive control) and tpbB/pMQ70-tpbB-Y95F, but not with tpbB/pMQ70 (negative control). (9.95 MB TIF) [file ppat.1000483.s005.tif]
