# Supplementary material for: Connecting Quorum Sensing, c-di-GMP, Pel Polysaccharide, and Biofilm Formation in Pseudomonas aeruginosa through Tyrosine Phosphatase TpbA (PA3885)
Source: PLoS Pathog. 2009 Jun 19;5(6):e1000483. doi: 10.1371/journal.ppat.1000483 (PMC2691606; doi:10.1371/journal.ppat.1000483)
Supplement: Table S1 — Partial list of induced genes in biofilm cells in LB medium after 4 and 7 h at 37°C for the tpbA mutant versus wild-type PA14 using three sets of DNA microarrays (note RNAlater was used for the 4 h set and the second set at 7 h). (0.07 MB DOC) [file ppat.1000483.s006.doc]

|  |  |  | **Fold change** | | |  |
| --- | --- | --- | --- | --- | --- | --- |
| **PAO1 ID** | **PA14 ID** | **Gene Name** | **4 h** | **7 h-1st** | **7 h-2nd** | **Descriptions** |
| EPS production | |  |  |  |  |  |
| PA3058 | PA14_24560 | *pelG* | 1.1 | -1.1 | 1.9 | Predicted membrane protein PelG |
| PA3059 | PA14_24550 | *pelF* | 1.5 | 1.1 | 4.0 | Glycosyltransferase PelF |
| PA3060 | PA14_24530 | *pelE* | 1.6 | 1.2 | 2.8 | Sucrose synthase PelE |
| PA3061 | PA14_24510 | *pelD* | 1.2 | 1.3 | 4.3 | L-lactate permease PelD |
| PA3062 | PA14_24500 | *pelC* | 1.3 | 1.3 | 3.5 | Glycosyltransferase PelC |
| PA3063 | PA14_24490 | *pelB* | 1.3 | 1.2 | 1.7 | Conserved hypothetical protein PelB |
| PA3064 | PA14_24480 | *pelA* | 1.7 | 1.5 | 4.0 | Oligogalacturonide lyase PelA |
| PA3145 | PA14_23460 | *wbpL* | 2.6 | -1.1 | 6.1 | Glycosyltransferase WbpL |
| PA3146 | PA14_23450 | *wbpK* | 11.3 | 3.2 | 1.5 | NAD-dependent epimerase/dehydratase WbpK |
|  |  |  |  |  |  |  |
| Posttranslational modification | | |  |  |  |  |
| PA3885 | PA14_13660 | *tpbA* | 42.2 | 17.1 | 119.4 | Protein tyrosine phosphatase |
|  |  |  |  |  |  |  |
| Transport of small molecules | | |  |  |  |  |
| PA3531 | PA14_18670 | *bfrB* | 2.1 | 3.0 | 1.3 | Bacterioferritin |
| PA4142 | PA14_10350 |  | 1.0 | 3.2 | 2.6 | Probable secretion protein |
| PA4143 | PA14_10340 |  | 1.1 | 2.5 | 3.2 | Probable toxin transporter |
| PA4144 | PA14_10330 |  | 1.0 | 1.7 | 2.6 | Probable outer membrane protein precursor |
|  |  |  |  |  |  |  |
| Others |  |  |  |  |  |  |
| PA1168 | PA14_49310 |  | 2.1 | 6.5 | 8.6 | Hypothetical protein |
| PA1169 | PA14_49300 |  | 1.4 | 3.2 | 6.5 | Probable lipoxygenase |
| PA2407 | PA14_33560 |  | 4.6 | 1.1 | 1.2 | Probable adhesion protein |
| PA2440 | PA14_33060 |  | 1.4 | 1.6 | 3.2 | Hypothetical protein |
| PA2441 | PA14_33050 |  | 1.5 | 3.2 | 34.3 | Hypothetical protein |
| PA3886 | PA14_13650 |  | 3.5 | 2.8 | 3.0 | Hypothetical protein |
| PA4139 | PA14_10380 |  | 5.3 | 22.6 | 7.0 | Hypothetical protein |
| PA4140 | PA14_10370 |  | 1.9 | 14.9 | 19.7 | Hypothetical protein |
| PA4624 | PA14_61190 |  | 2.0 | 2.1 | 4.0 | Putative hemolysin activation/secretion protein |
| PA4625 | PA14_61200 |  | 2.8 | 3.7 | 4.9 | Putative adhesin/hemagglutinin protein |
