# Supplementary material for: Connecting Quorum Sensing, c-di-GMP, Pel Polysaccharide, and Biofilm Formation in Pseudomonas aeruginosa through Tyrosine Phosphatase TpbA (PA3885)
Source: PLoS Pathog. 2009 Jun 19;5(6):e1000483. doi: 10.1371/journal.ppat.1000483 (PMC2691606; doi:10.1371/journal.ppat.1000483)
Supplement: Table S2 — Partial list of repressed genes in biofilm cells in LB medium after 4 and 7 h at 37°C for the tpbA mutant versus wild-type PA14 using three sets of DNA microarrays (note RNAlater was used for the 4 h set and the second set at 7 h). (0.10 MB DOC) [file ppat.1000483.s007.doc]

|  |  |  | **Fold change** | | |  |
| --- | --- | --- | --- | --- | --- | --- |
| **PAO1 ID** | **PA14 ID** | **Gene Name** | **4 h** | **7 h-1st** | **7 h-2nd** | **Descriptions** |
| Amino acid biosynthesis and metabolism | | | | |  |  |
| PA1838 | PA14_10550 | *cysI* | -1.3 | -2.8 | -4.0 | Sulfite reductase |
| PA4442 | PA14_57710 | *cysN* | -1.2 | -3.5 | -4.3 | ATP sulfurylase GTP-binding subunit/APS kinase |
| PA4443 | PA14_57720 | *cysD* | -1.1 | -4.0 | -3.5 | ATP sulfurylase small subunit |
| PA0281 | PA14_03670 | *cysW* | -1.1 | -3.0 | -3.7 | Sulfate transport protein CysW |
|  |  |  |  |  |  |  |
| Transport of small molecules | | |  |  |  |  |
| PA2202 | PA14_36230 |  | 1.1 | -7.0 | -4.0 | Putative amino acid transport system permease |
| PA2204 | PA14_36200 |  | -1.3 | -5.3 | -6.1 | Probable binding protein component of ABC transporter |
| PA2329 | PA14_34500 |  | -1.2 | -2.5 | -3.7 | Probable ATP-binding component of ABC transporter |
| PA0283 | PA14_03700 | *sbp* | -1.1 | -3.2 | -4.0 | Sulfate-binding protein precursor |
|  |  |  |  |  |  |  |
| Protein secretion/export apparatus | | |  |  |  |  |
| PA4302 | PA14_55890 | *tadA* | 1.0 | -3.0 | -3.5 | Probable type II secretion system protein |
| PA4304 | PA14_55920 | *rcpA* | 1.0 | -3.2 | -3.0 | Probable type II secretion system protein |
|  |  |  |  |  |  |  |
| Motility & attachment | |  |  |  |  |  |
| PA0173 | PA14_02180 | *cheB* | -1.1 | -1.5 | -2.3 | Probable methylesterase |
| PA0174 | PA14_02190 |  | -1.5 | -2.1 | -2.0 | Probable chemotaxis protein |
| PA0175 | PA14_02200 |  | -1.5 | -2.1 | -1.6 | Probable chemotaxis protein methyltransferase |
| PA0176 | PA14_02220 | *aer2* | -1.9 | -2.1 | -2.0 | Aerotaxis transducer |
| PA0177 | PA14_02230 | *cheW* | -1.6 | -2.5 | -1.7 | Probable purine-binding chemotaxis protein |
| PA0178 | PA14_02250 | *cheA* | -2.0 | -2.5 | -2.1 | Probable two-component sensor |
| PA0179 | PA14_02260 |  | -2.1 | -2.8 | -1.9 | Probable two-component response regulator |
| PA0180 | PA14_02270 |  | -1.5 | -1.4 | -1.2 | Probable chemotaxis transducer |
| PA1097 | PA14_50220 | *fleQ* | -1.4 | -1.1 | -1.4 | Transcriptional regulator FleQ |
| PA1099 | PA14_50180 | *fleR* | -1.4 | -1.6 | -1.6 | Two-component response regulator |
| PA4305 | PA14_55930 | *rcpC* | 1.1 | -3.0 | -3.2 | Putative pilus assembly protein |
| PA4306 | PA14_55940 | *flp* | -1.4 | -3.7 | -4.6 | Putative pilus assembly protein |
| PA4307 | PA14_55960 | *pctC* | -2.3 | -2.1 | -2.6 | Chemotactic transducer PctC |
| PA4310 | PA14_56010 | *pctB* | -1.5 | -1.5 | -1.7 | Chemotactic transducer PctB |
|  |  |  |  |  |  |  |
| Virulence factors | |  |  |  |  |  |
| PA1246 | PA14_48115 | *aprD* | -1.9 | -1.6 | -2.0 | Alkaline protease secretion protein AprD |
| PA1247 | PA14_48100 | *aprE* | -1.4 | -1.4 | -2.0 | Alkaline protease secretion protein AprE |
| PA1248 | PA14_48090 | *aprF* | -1.4 | -1.5 | -2.1 | Alkaline protease secretion protein AprF |
| PA1249 | PA14_48060 | *aprA* | -2.1 | -1.2 | -1.9 | Alkaline metalloproteinase precursor |
| PA1250 | PA14_48040 | *aprI* | -1.4 | 1.0 | -1.4 | Alkaline proteinase inhibitor AprI |
| PA1871 | PA14_40290 | *lasA* | -2.0 | -1.1 | -1.1 | LasA protease |
| PA3724 | PA14_16250 | *lasB* | -1.7 | 1.1 | 1.0 | LasB elastase |
|  |  |  |  |  |  |  |
| Others |  |  |  |  |  |  |
| PA0284 | PA14_03710 |  | -1.4 | -4.3 | -4.3 | Hypothetical protein |
| PA1849 | PA14_40610 |  | -1.2 | -13.0 | -1.2 | Hypothetical protein |
| PA1914 | PA14_39780 |  | 1.0 | -3.7 | -4.0 | Putative halovibrin |
| PA2062 | PA14_30440 |  | -1.2 | -3.0 | -3.5 | Probable pyridoxal-phosphate dependent enzyme |
| PA3441 | PA14_19590 |  | 1.1 | -3.7 | -4.3 | Probable molybdopterin-binding protein |
| PA3444 | PA14_19560 |  | -1.1 | -3.0 | -3.2 | Putative sulfonate monooxygenase |
| PA3931 | PA14_13010 |  | -1.2 | -2.6 | -3.5 | Conserved hypothetical protein |
| PA5024 | PA14_66420 |  | -1.1 | -2.8 | -3.5 | Putative membrane protein |
