# Supplementary material for: Connecting Quorum Sensing, c-di-GMP, Pel Polysaccharide, and Biofilm Formation in Pseudomonas aeruginosa through Tyrosine Phosphatase TpbA (PA3885)
Source: PLoS Pathog. 2009 Jun 19;5(6):e1000483. doi: 10.1371/journal.ppat.1000483 (PMC2691606; doi:10.1371/journal.ppat.1000483)
Supplement: Table S3 — Primers used in this study. (0.05 MB DOC) [file ppat.1000483.s008.doc]

| **Name** | **Sequence (5' to 3')** | **Description** |
| --- | --- | --- |
| PA14_13660-VF | GGAGTGCTCGGGGTCCTTTA | Verification of *tpbA* tn insertion |
| PA14_13660-VR | ACGACGATGCGGTACATGGC | Verification of *tpbA* tn insertion |
| GB-3a | TACAGTTTACGAACCGAACAGGC | Verification of *tpbA* tn insertion |
| R1 | ATCGACCCAAGTACCGCCAC | Verification of *tpbA* tn insertion |
| PA14_13660-F1-NheI | GCCCCCGCTAGCAAGAAGGAGATATACCATGCACCGTTCACCGCTCGCCTGG | Construction of pMQ70-*tpbA* |
| PA14_13660-R-cHis-HindIII | GCCCCCAAGCTTCTAGTGGTGGTGGTGGTGGTGCTCGAGCGGCCGGTCGAGCGCCTGGGC | Construction of pMQ70-*tpbA* |
| PA14_49890-F1-NheI | GCCCCCGCTAGCAAGAAGGAGATATACCCGTGATGAACCGTCGTCGTCGCTAT | Construction of pMQ70-*tpbB* |
| PA14_49890-R-cHis-HindIII | GCCCCCAAGCTTCTAGTGGTGGTGGTGGTGGTGCAGCAGGCTGCCGCCGGAGCCTTTC | Construction of pMQ70-*tpbB* |
| PA14_49890seq-F | GTCCTGTGCCTGCTGCTTAC | Sequencing pMQ70-*tpbB* |
| PA14_49890seq-R | AACAGTACCGCCAATTGCTC | Sequencing pMQ70*-tpbB* |
| pMQ70-F | GCGTCACACTTTGCTATGCCATAGC | Sequencing pMQ70 derivatives |
| pMQ70-R | CTACTGCCGCCAGGCAAATTCTGTTT | Sequencing pMQ70 derivatives |
| PA14_13660-F-NdeI | GGGAATTCCATATGCACCGTTCACCGCTC | Construction of pET28b-13660n |
| PA14_13660-R-BamHI | GCCCCGGATCCTCGGCCCTGCGCGTGTTTA | Construction of pET28b-13660n |
| PA14_13660-F-*Xba*I | GCCCCTCTAGAAAGAAGGAGATATACCATGCACCGTTCACCGCTCGCCTGG | Construction of pET28b-13660c |
| PA14_13660-R-*Xho*I | GCCCCCTCGAGCGGCCGGTCGAGCGCCTGGGCCATC | Construction of pET28b-13660c |
| LuxAB inside | CTTTCAATTTCCGCTTTCAAGC | Identification of transposon insertion |
| LuxAB outside | CGATGGTGAGTTGTTCAAAATC | Identification of transposon insertion |
| Arb1 | GGCCACGCGTCGACTAGTACNNNNNNNNNNGATAT | Identification of transposon insertion |
| Arb2 | GGCCACGCGTCGACTAGTAC | Identification of transposon insertion |
| T7 promoter | TAATACGACTCACTATAGGG | Sequencing pET28b derivatives |
| T7 terminator | GCTAGTTATTGCTCAGCGG | Sequencing pET28b & pGEM-T easy derivatives |
| SP6 | GATTTAGGTGACACTATAG | Sequencing pGEM-T easy derivatives |
| *pelA*-F | CCTGGAACAGCCAGGTAATG | RT-PCR |
| *pelA*-R | AAACCGCGATTGAAAAACAG | RT-PCR |
| PA4139-F | GATGTGGTCAAGGTGTCCAAC | RT-PCR |
| PA4139-R | TCAGGTCCACTCAGTGTCGTAG | RT-PCR |
| PA4625-F | ATCCGTTCATCAACCAGAGC | RT-PCR |
| PA4625-R | CACCAGGTAGTCCGACGAGT | RT-PCR |
| pPA14_13660-F-HindIII | GCCCCAAGCTTGCGGGGTGGTCCGCTTCGGCGGCCT | Construction of pLP-p*tpbA* plasmid |
| pPA14_13660-R-BamHI | GCCCCGGATCCGGCGGAGCCAGGCGAGCGGTGAACG | Construction of pLP-p*tpbA* plasmid |
| pLP170-MCS-F | CCTATAAAAATAGGCGTATCACGAGG | Sequencing for pLP-p*tpbA* plasmid |
| pLP170-MCS-R | GGTCATAGCTGTTTCCTGTGTGATAA | Sequencing for pLP-p*tpbA* plasmid |
| PA14_49890_Y48F-F | CCTGCGCGCCTTTGCGGACCCCAAC | Site-directed mutagenesis for Y48F of TpbB |
| PA14_49890_Y48F-R | GTTGGGGTCCGCAAAGGCGCGCAGG | Site-directed mutagenesis for Y48F of TpbB |
| PA14_49890_Y62F-F | GTTCCATCAGCTTTACCGTCGAAGC | Site-directed mutagenesis for Y62F of TpbB |
| PA14_49890_Y62F-R | GCTTCGACGGTAAAGCTGATGGAAC | Site-directed mutagenesis for Y62F of TpbB |
| PA14_49890_Y95F-F2 | GCGCCATCGTCTTCGACCGCCAGGG | Site-directed mutagenesis for Y95F of TpbB |
| PA14_49890_Y95F-R2 | CCCTGGCGGTCGAAGACGATGGCGC | Site-directed mutagenesis for Y95F of TpbB |
